# A Review of Strategies for the Synthesis of N-Doped Graphene-Like Materials

**DOI:** 10.3390/nano10112286

**Published:** 2020-11-18

**Authors:** Alenka Vesel, Rok Zaplotnik, Gregor Primc, Miran Mozetič

**Affiliations:** Department of Surface Engineering, Jozef Stefan Institute, Jamova cesta 39, 1000 Ljubljana, Slovenia; rok.zaplotnik@ijs.si (R.Z.); gregor.primc@ijs.si (G.P.); miran.mozetic@ijs.si (M.M.)

**Keywords:** graphene, carbon nanowalls, nitrogen doping, plasma synthesis, material characterization, X-ray photoelectron spectroscopy (XPS), Raman spectroscopy, defects

## Abstract

Methods for synthesizing nitrogen-doped graphene-like materials have attracted significant attention among the scientific community because of the possible applications of such materials in electrochemical devices such as fuel cells, supercapacitors and batteries, as well as nanoelectronics and sensors. The aim of this paper is to review recent advances in this scientific niche. The most common synthesis technique is nitridization of as-deposited graphene or graphene-containing carbon mesh using a non-equilibrium gaseous plasma containing nitrogen or ammonia. A variety of chemical bonds have been observed, however, it is still a challenge how to ensure preferential formation of graphitic nitrogen, which is supposed to be the most favorable. The nitrogen concentration depends on the processing conditions and is typically few at.%; however, values below 1 and up to 20 at.% have been reported. Often, huge amounts of oxygen are found as well, however, its synergistic influence on N-doped graphene is not reported. The typical plasma treatment time is several minutes. The results reported by different authors are discussed, and future needs in this scientific field are summarized. Some aspects of the characterization of graphene samples with X-ray photoelectron spectroscopy and Raman spectroscopy are presented as well.

## 1. Introduction

**Figure 1:**
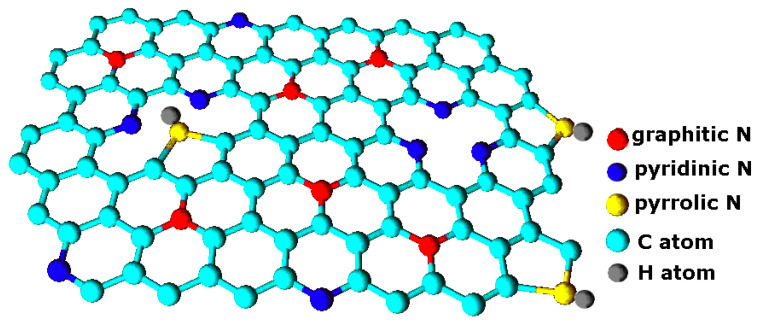
The most common bonding configuration of nitrogen atoms in graphene.

Graphene is a promising material for future applications in electrochemical devices. It can be in the form of a single or multilayer film or as vertically oriented graphene sheets, which are often called carbon nanowalls (CNWs). The high capacitance enabled by the large specific surface area of CNWs makes them useful for supercapacitors [1]. The capacitance may be further altered by the introduction of heteroatoms such as O and N [1]. Graphene can also be used for fuel cells, field emitters and batteries [2,3]. In all cases, the electrical properties of the graphene are very important. Both the electrical and chemical properties of graphene can be changed by nitrogen doping. Therefore, the synthesis of N-doped graphene has become a significant scientific challenge in the last few years. Furthermore, N-doped graphene materials are also crucial for the development of metal-free carbon-based catalysts for application in fuel cells [4]. The synthesis of vertically-oriented graphene sheets (CNWs) or similar structures is often performed by plasma-enhanced chemical vapor deposition (PECVD) in CH_4_ (or, rarely, in other gaseous hydrocarbons) atmosphere with the presence of other reactive gases that may enhance the quality of CNWs. A review of PECVD methods for CNW deposition was published recently in [5]. However, the incorporation of nitrogen into CNWs and the other graphene-like structures is still an important task, especially during the growth of the sample (direct synthesis), which would enable the homogenous distribution of nitrogen in the deposit. Nowadays, nitrogen is often doped by post-treatment of graphene-like materials in plasmas containing nitrogen; however, this may lead to nitrogen concentrated preferentially at the surface of the graphene-containing film. The conductivity of N-doped graphene depends on the concentration of nitrogen and its chemical binding [6]. Therefore, the characterization of nitrogen peak in X-ray photoelectron spectroscopy (XPS) spectra is crucial. The most common nitrogen configurations that are usually found in N-doped carbon nanoparticles are pyridinic, pyrrolic and quaternary (graphitic) nitrogen [6,7,8]. Schematically they are shown in Figure 1. For obtaining the enhanced electrical properties, graphitic nitrogen is essential [9,10]; however, other nitrogen configurations are also always present, and their concentration may significantly alter the physical and chemical properties of N-doped graphene. Therefore, the optimal procedure for obtaining N-doped graphene should enable the formation of more graphitic and less pyridinic and pyrrolic nitrogen, which may even reduce the conductivity [11,12]. For graphitic nitrogen configuration, n-type doping effect was reported, whereas pyridinic and pyrrolic nitrogen lead to p-type doping effect [13].

In this paper we give a review on one- or two-step synthesis procedures of N-doped graphene-like materials by plasma techniques. For comparison, also some classical chemical methods are summarized. Furthermore, in Appendix A we report about the peculiarities of application of Raman spectroscopy for characterization of graphene-like materials, whereas in Appendix B problems and challenges of XPS characterization of graphene-like materials are given.

## 2. Plasma-Assisted Synthesis of N-Doped Graphene

### 2.1. One-Step Plasma Deposition Procedures

Only a few authors have reported direct plasma syntheses of N-doped graphene layers and CNWs. Bundaleska et al. [14] performed a direct single-step synthesis of N-doped graphene using an atmospheric pressure microwave plasma created in ethanol and ammonia with Ar as a carrier gas at 2 kW. The nitrogen configuration in the material was additionally altered by applying infrared (IR) and ultraviolet (UV) radiation. The relative amount of nitrogen N/C was 0.004. The ratio of various nitrogen sub-peaks corresponding to different configurations depended on whether the sample was exposed to radiation or not. The relative amount of pyridinic and pyrrolic nitrogen was higher for the irradiated samples.

Tatarova et al. [15] used a powerful microwave discharge at atmospheric pressure for graphitization and formation of nitrogen-doped graphene sheets. Argon, ethanol, and nitrogen were used as processing gases. The discharge power was varied between 500 and 2000 W. The gas temperature was estimated to as high as 4000 K. The precursors partially decomposed upon high temperature, and the radicals condensate in the flowing afterglow to form clusters of graphene flakes. The authors also applied soft UV radiation to kick off the epoxy oxygen groups and sp^3^ carbons. If nitrogen in the processing gas was absent, the sp^2^ bonds in graphene at 284.4 eV and the sp^3^ at 285.2 eV were found using asymmetric fit parameters for C1s obtained on HOPG. The ether and hydroxyl groups were found as well at 286.3 eV and a broad shake-up feature at 290.6 eV. When nitrogen was added, only symmetric fitting was used, and the C-N groups were found at 285.7 eV, the epoxy and carbonyl groups at 287.6 eV, and carboxylate groups at 289 eV. The concentration of nitrogen in the graphene sheaths was 0.2 at.% and oxygen 8 at.%. The authors concluded that the XPS undoubtedly confirmed the doping of graphene by nitrogen atoms, mainly in pyridinic form.

Boas et al. [8] also used a microwave discharge and managed to synthesize thin films of N-doped graphene at low pressure. They were able to sustain plasma at voltage as low as 85 V and the pressure of 43 Torr using a 500-W discharge power. Different concentrations of nitrogen and methane were adopted. The treatment time was 150 s, and the substrate temperature 760 °C. The XPS C1s peaks were fitted with three symmetric sub-peaks, i.e., the main peak at 284.6 eV assigned to sp^2^ hybridized carbon, as well as α-sp^2^ at 286 eV and α-sp^3^ at 288.5 eV, reflecting the binding of O and N atoms to sp^2^ C and sp^3^ C, respectively. The XPS N1s peaks were fitted with two sub-peaks, i.e., 398.4 eV corresponding to pyridinic nitrogen and at 397.5 eV corresponding to another type of nitrogen defects. The experimental results were compared to simulations to conclude the chemical state changes in the graphene structure. The concentration of nitrogen was between 0.2 and 4.2 at.%.

Low-pressure gaseous plasma sustained in H_2_/CH_4_/N_2_ gases was also used by Terasawa and Saiki [16] for deposition of graphene islets on copper substrates, but the experimental details were not provided. Despite the weak XPS N1s peak, the authors managed to conclude that the majority of nitrogen was incorporated in the graphitic sites. Also Kumar et al. [17] used H_2_/CH_4_/N_2_ plasma excited by microwaves at low pressure. The pressure was 10 Torr and the discharge power up to 400 W. The treatment time of 4–5 min enabled the deposition of nitrogen-doped few-layer graphene film. The nitrogen content, as deduced from XPS survey spectra, was about 2 at.%. The deconvolution of the N1s spectra revealed that the majority of nitrogen was in graphitic form.

### 2.2. Plasma Post-Treatment Procedures

As it will be shown in this section, N-doped graphene samples are mostly prepared by using a two-step procedure, including: (1) synthesis of graphene-like material by using mostly plasma-enhanced chemical vapour deposition (PECVD) as a first step, and (2) post-treatment of as-deposited materials with nitrogen-containing plasma, such as N_2_ or NH_3_ to obtain N-doped graphene-like structures. The second step is often performed in-situ just after accomplishing the first step.

Evlashin et al. [18] performed post-plasma treatment of CNWs in DC glow discharge in N_2_ plasma with the pressure below 3 Pa. The modification was performed on a time scale of 2 h. Such modification led to the incorporation of 3 at.% of nitrogen (and also almost 30 at.% of oxygen). N-doped CNWs were found useful for supercapacitor fabrication. In another paper, Evlashin et al. [1] used DC plasma created in O_2_, N_2_, or their mixtures to investigate the mechanisms that lead to the increased specific capacitance. Four at.% of nitrogen and ten at.% of oxygen was found in CNWs. Oxygen-plasma treated CNWs exhibited higher specific capacitance (8.9 F/cm^3^) than those treated in nitrogen (4.4 F/cm^3^).

Cho et al. [12] investigated the electrical conductivity of CNWs after post-plasma treatment in N_2_ plasma sustained with a capacitively coupled radio-frequency discharge (CCP). Treatment time was varied between 30 and 300 s. They found increased electrical conductivity after 30 s of treatment. However, with further increasing of treatment time, the electrical conductivity decreased. They found that that carrier density was decreasing with treatment time, whereas the carrier mobility was increasing. This was correlated with an increase of the nitrogen content and density of defects in CNWs, followed by their decrease with increasing treatment time. Nitrogen to carbon ratio increased from 0.095 at 30 s to 0.22 at 300 s. No remarkable changes in surface morphology of CNWs after nitrogen plasma treatment were observed.

Singh et al. [11] used a post-plasma treatment procedure using NH_3_ gas. They treated graphene oxide structures in NH_3_ plasma for various treatment times and found the best properties at short treatment times up to 5 min, where an increase of sp^2^ carbon occurred, and graphitic nitrogen prevailed, leading to the n-type conductivity. Prolonged plasma treatment caused a decrease of sp^2^ carbon and a decrease of electrical conductivity, which was explained by the high concentration of pyridinic nitrogen; however, graphitic nitrogen still persisted. Nitrogen content was increasing with treatment time up to N/C = 0.25. For 5 min treatment, N/C was 0.15. In general, the time evolution of functional groups was as follows: at the initial stages, pyrrolic nitrogen was formed with a small contribution of pyridinic and graphitic. After 5 min of treatment, a significant increase of graphitic content occurred, whereas pyrrolic nitrogen saturated. For long treatments, pyridinic nitrogen significantly increased together with a continuous increase of graphitic nitrogen. Oxygen content did not depend much on treatment time and the O/C ration was up to 0.27; however, the smallest ratio of 0.15 was found for a treatment time of 5 min.

McClure et al. [19] performed plasma-post treatment of CNWs in N_2_/Ar low-pressure plasma created at various RF powers. Nitrogen concentration after doping was 4–20 at.%, depending on the power used. Significant amounts of oxygen (39–52 at.%) were found as well. With the increasing RF power, a shift of XPS low-binding energy N1s peaks (398.7–400.3 eV) towards high-binding energy N1s peaks (~401–404 eV) occurred. The results of Raman spectroscopy showed that *I*_D_/*I*_G_ ratio of non-doped CNWs was higher if they were synthesized at a higher temperature, indicating the formation of smaller crystallite sizes and grains. After N-doping, the change in the *I*_D_/*I*_G_ ratio was less pronounced for CNWs with the smaller crystallite size.

Yen et al. [20] performed in-situ doping of CNWs in NH_3_ plasma immediately after accomplishing their synthesis. Interestingly, and opposite to what has been observed by other authors, only sp^2^ C-N (pyridinic) nitrogen was found, whereas other nitrogen configurations such as pyrrolic and graphitic were not observed. Nitrogen concentration was about 7.8 at.%.

Zhao et al. [21] used plasma treatment of vertically-oriented graphene nanowalls in the PECVD reactor with NH_3_ plasma. The substrate was heated to 350 °C, and the sample was treated for 30 min. They found a small amount of nitrogen at about 1.2 at.%. Nitrogen was in the form of amino groups because only one peak at 399.6 eV was found in the XPS spectrum. Oxygen, which was present already on pristine graphene, was reduced for 7.4% after the NH_3_-plasma treatment. They found enhanced field emission properties of N-doped samples.

Achour et al. [22] treated CNWs in the PECVD reactor using Ar/N_2_ or Ar/O_2_ plasma. The same reactor was first used to synthesize pristine CNWs; however, because of a rather high pressure of residual atmosphere (about 100 Pa), already virgin CNWs contained significant concentration of nitrogen (5.1 at.%) as well as oxygen (8.7 at.%). After the plasma treatment in Ar/N_2_ or Ar/O_2_ mixture, nitrogen concentration was increased to 12.4 and 13.5 at.%, respectively, and oxygen to 23.5 and 33.9 at.%, respectively. Both plasma treatments caused an increased concentration of pyrrolic nitrogen, especially Ar/N_2_. SEM images of N-doped CNWs showed more branched CNWs, especially in the case of oxygen plasma treatment, which was explained by etching of the CNWs.

Jeong et al. [23] synthesized N-doped graphene for ultracapacitors with 4-times larger capacitance than for pristine graphene. They used the PECVD reactor first to reduce graphene oxide in H_2_ plasma to graphene, followed by subsequent N_2_ plasma treatment that induced 1.7 to 2.5 at.% of nitrogen in the form of pyridinic, pyrrolic, and graphitic nitrogen. Improved capacity was correlated with a certain N-configuration at basal planes. Oxygen was found as well in concentrations of about 16–25 at.%.

McManus et al. [24] employed two-step remote plasma treatments in Ar, followed by NH_3_/H_2_ treatment to form n-type N-doped graphene. Argon plasma was used to make defects first (vacancy defects rather than edge defect), which were then healed with NH_3_/H_2_-plasma treatment to incorporate nitrogen. Nitrogen content was 2.5 at.%, and 7% of this nitrogen was in graphitic form, which contributed to the n-doping of the sample. Raman results showed a significant increase of D component and decrease and broadening of 2D after Ar treatment. When NH_3_/H_2_ was applied, the D peak was reduced, and 2D slightly recovered. The shifts of Raman 2D and G peaks, which are an indicator of doping, were found to be shifted to higher values after Ar treatment (increased p-doping), whereas after further NH_3_/H_2_ treatment, they were shifted back to lower values (increased n-doping). A similar synthesis was also performed by McEvoy et al. [19]; however, they used first remote O_2_ plasma treatment followed by further NH_3_/H_2_ treatment. The authors were mostly focused on defects induced by O_2_ plasma treatment for various treatment times, and results for NH_3_/H_2_ treatment are only briefly mentioned. Nitrogen content was about 3%.

Lin et al. [25] applied ECR enhanced MW nitrogen plasma source in two different modes of operation, i.e., ion- or atom-mode. In the latter case, a metal ion trap was used to remove ions and allow only neutral atoms to react with graphene. Ion and neutral-atom fluxes were 4 × 10^12^ s^−1^ cm^−2^ and 2.5 × 10^15^ s^−1^ cm^−2^, respectively. The working pressure was 5 × 10^−5^ mbar. When exposed to ions, n-type was formed attributed to graphitic nitrogen. When exposed to atoms, pyridinic nitrogen prevailed, causing only minor n-doping. Annealing the sample at 850 °C helped in removing weak nitrogen adsorbates that may have a negative effect on the electronic structure.

Zeng et al. [26] used RF N_2_ plasma treatment at various powers from 30 to 70 W to tune the properties of N-doped CNWs. The n-type doping was obtained, and only graphitic nitrogen was found, whose concentration was increasing with increasing discharge power. Electron concentration was increasing as well. Because of increased disorder and degraded crystallinity, *I*_D_/*I*_G_ ratio was also increasing with increasing RF power.

Baraket et al. [27] investigated the functionalization of graphene with NH_3_ plasma created at various pressures for DNA detection. NH_3_ pressure was varied between 25 to 90 mTorr. Nitrogen concentration was increasing with increasing pressure from 5 (at 25 mTorr) to 20 at.% (at 90 mTorr). Raman D-peak intensity was also increasing with the pressure. The presence of amino groups was proven by XPS chemical derivatization, and 45% of nitrogen was assigned to amino groups. Amine functionalized graphene showed enhanced sensitivity for DNA detection.

Lee et al. [28] used an atmospheric-pressure plasma jet (APPJ) to treat mono or multilayer graphene. Treatment time and a distance of the APPJ jet to the sample surface was varied. N_2_ was used as a feed gas. To prevent the mixing of the surrounding atmosphere with the jet, APPJ was closed in a box filled with N_2_. After the treatment, the surface became hydrophilic, as the water contact angle dropped from 84° to 44°. Nitrogen was mostly in pyridinic configuration, although other configurations were present as well (amine, pyrrolic, graphitic). Thinner graphene layers were more prone to modification because a higher increase of *I*_D_/*I*_G_ was observed. *I*_D_/*I*_G_ was increasing with increasing treatment time and with decreasing distance of APPJ to the sample.

Santhosh et al. [29] compared the doping of CNWs treated in N_2_ or NH_3_ plasma. A source of nitrogen radicals was inductively coupled RF plasma, and the samples were placed in the afterglow region. Samples were treated for different times up to 40 s or 25 s for N_2_ or NH_3_ plasma, respectively. To prevent heating, they were treated in pulses. As revealed from SEM, N_2_-plasma afterglow caused etching of CNWs, that was not observed for NH_3_. The maximum nitrogen content was 8.0 and 2.8 at.% for N_2_ and NH_3_, respectively. All three nitrogen configurations were found; however, for NH_3_ plasma, also amino groups were found, whereas for N_2_, some oxygenated nitrogen species were observed. *I*_D_/*I*_G_ ratio was almost linearly decreasing with increasing treatment time in N_2_ plasma. In the case of NH_3_ plasma, they found that after the initial decrease of *I*_D_/*I*_G_ with treatment times up to 15 s, the ratio *I*_D_/*I*_G_ increased at longer treatment times. A decrease in electrical conductivity was observed as a result of afterglow treatments, especially when plasma was sustained in N_2_. High nitrogen concentrations (above 5 at.%) were not found beneficial for conductivity.

Manojkumar et al. [30] used nitrogen ion implantation at 2 kV for 10, 20, and 30 min to induce defects in CNWs. The samples were biased (negative pulsed DC voltage) during implantation. The source of nitrogen ions was RF plasma. Nitrogen was implanted at the CNW edges and also a few nanometers within the samples. This caused the formation of defects as observed by Raman spectroscopy and revealed from a decrease of *I*_D_/*I*_G_ and *I_2_*_D_/*I*_G_ ratio. Depending on the treatment time, the nitrogen concentration was between 7.6 to 8.8 at.%. Oxygen (~13 at.%) was also present. Nitrogen was deconvoluted to three peaks, which were attributed to lone-pair localized nitrogen (N1) at 309.6 eV (pyridinic/pyrrolic/nitrile), lone-pair delocalized at 400.9 eV (N2), and quaternary nitrogen at 402.6 eV (N3). The concentration of nitrogen in the N1 configuration was increasing with treatment time, whereas the concentration of N2 and N3 was decreasing. Nevertheless, N1 was always dominating. A significant transformation of sp^2^ carbon to sp^3^ was observed after nitrogen implantation, what was explained by defect formation. No noticeable changes in the morphology of CNWs were observed after 20 min of implantation; however, extensive sputtering was observed for the sample treated for 30 min.

The literature about post-deposition plasma treatment indicates that different authors have used plasmas sustained in nitrogen or ammonia. Sometimes also an addition of another gas was used. Most authors used low-pressure high-frequency discharges to sustain the gaseous plasma. The discharge parameters varied significantly, and a few authors also stated the fluxes of reactive species. The surface finish should depend on the fluxes. The variation of the fluxes with discharge parameters is complex because numerous details may be important. For example, the density of reactive gaseous species depends on the type of discharge, the discharge coupling and power, the pressure, the gas purity, the geometry of the plasma reactor etc. Taking into account all these effects, it is difficult to draw general conclusions based on comparison or surface finish reported by different authors. One observation that is common among all authors is that ammonia plasma will favourite functionalization with amino groups. In contrast, nitrogen plasma treatment will result in the formation of various other nitrogen-containing functional groups. A few authors also mention etching of nano-carbon materials during exposure to either ammonia or nitrogen plasma. The etching should be caused by sputtering when the samples are negatively biased, but chemical etching should prevail in cases where samples were left at a floating potential. Obviously, chemical etching only occurs upon exposure to afterglows.

## 3. Non-Plasma Synthesis of N-Doped Graphene

Generally, non-plasma synthesis procedures are often used by authors, comprising mostly chemical vapor deposition (CVD) using various chemical precursors acting as a source of carbon and nitrogen. Opposite to post-plasma treatment techniques described above, non-plasma CVD techniques enable a direct synthesis of N-doped graphene. Some of them are mentioned below.

Usachov et al. [9] proposed a CVD method for the growth of N-doped graphene from s-triazine precursor. The concentration of nitrogen in graphene was 1–2 at.%. Nitrogen was mostly in the pyridinic form. Therefore, they developed an additional procedure to convert nitrogen to graphitic form. This was done with the help of Au intercalation, followed by annealing up to 635° for 30 min.

Deng et al. [31] synthesized N-doped graphene via the reaction of tetrachloromethane with lithium nitride. Nitrogen content N/C was in the range of 4.5–16.4%. For the sample containing low nitrogen content, graphitic nitrogen prevailed, whereas for the sample with a high nitrogen concentration, pyridinic and pyrrolic nitrogen dominated. The authors also investigated the thermal stability of N-doped graphene at 600 °C. The nitrogen concentration was slightly reduced; however, a still significant amount of nitrogen was retained, indicating that nitrogen species were rather thermally stable. The most thermally unstable was pyrrolic nitrogen. Results of Raman spectroscopy showed a shift of G band of N-doped graphene with a low N content to the lower frequency (typical for n-type), whereas for the sample with a high N content to the higher frequency (typical for p-type), implying different doping effect. They concluded that graphitic nitrogen, as found in the sample with a low N content, is the n-type dopant, whereas pyridinic and pyrrolic found at higher N concentration are p-type dopants. Contrarily, Lu et al. [32] observed transformation from p-type N-doped graphene to n-type, when nitrogen content was increased from 2.1 to 5.6%. They used CVD of 1,3,5-triazine to prepare N-doped samples. They found that pyridinic and pyrrolic N plays an important role in the transport behavior of carriers.

Qu et al. [4] used CVD to prepare N-doped graphene from CH_4_ and NH_3_ precursors for the application as electrocatalysts for oxygen reduction reaction (ORR) in fuel cells. Nitrogen (4 at.%) was in the form of two configurations (pyridinic and pyrrolic), both important for the ORR process. A very low *I*_D_/*I*_G_ ratio was obtained by Raman spectroscopy for N-doped samples (0.06–0.25), indicating high crystallinity. The samples showed better characteristics than Pt/C electrode. Also, Amano et al. [33] synthesized N-doped nanographene for ORR in polymer electrolyte fuel cells. However, they used in-liquid plasma created in a mixture of ethanol and iron phthalocyanine. Nitrogen content was 6 or 11 at.%, depending on the solvent used to disperse phthalocyanine. Four different nitrogen configurations were found: pyridinic, Fe-N, pyrrolic, and graphitic. The D to G ratio *I*_D_/*I*_G_ was 1.66 or 1.25, and the estimated grain size *L*_a_ = 15.3 or 11.6 nm, depending on the solvent used. Samples showed high catalytic activity, which can also be attributed to the presence of Fe-N bonds.

Opposite to other authors, who found nitrogen in various concentrations, Luo et al. [34] synthesized single-layer graphene doped with pure pyridinic nitrogen only. The deposition was performed on Cu substrate at 900 °C by CVD in H_2_ and C_2_H_4_ with the presence of NH_3_. The authors found that pyridinic nitrogen, which is often regarded to be important for ORR activity, is not an efficient stimulant for ORR.

Another approach to control the nitrogen configuration was made by Sui et al. [35]. The authors performed CVD synthesis of N-doped graphene from NH_3_ and CH_4_ gases at various temperatures from 880 to 1050 °C. They found that nitrogen concentration was decreasing with increasing temperature from 4.5 at.%, obtained at 880 °C, to only 0.7 at.%, at 1050 °C. The authors also observed linear relation between the XPS N1s peak position and the temperature. At the highest temperature, the N1s peak was positioned at 397.7 eV, corresponding to pyridinic N, whereas at the lowest temperature, the N1s peak was found at 400.2 eV, belonging to pyrrolic N. Effect of the growth temperature during the synthesis of N-doped graphene by free-radical reaction using pentachloropyridine on the nitrogen configuration was also investigated by Zhang et al. [13]; however, the authors used much lower temperatures, i.e., 230–600 °C. The synthesized films were dominated either with graphitic nitrogen (230–300 °C) or pyrrolic nitrogen (400–600 °C). The sample with graphitic-N configuration exhibited strong n-type doping and much higher electron mobility than for the sample with pyrrolic N. Also, Wei et al. [36] managed to synthesize graphene with dominant graphitic nitrogen by CVD in NH_3_ and CH_4_ at a temperature of 800 °C.

Although we can find more such publications about the chemical synthesis of N-doped graphene, we can notice from the papers mentioned above, that similar as for plasma procedures, also in the case of chemical synthesis, there is a problem of controlling nitrogen concentration, its configuration, and thus properties of N-doped graphene. However, there are few reports where authors obtained mostly one nitrogen configuration, or they were using temperature to manipulate the nitrogen configuration. The influence of temperature was not performed yet for plasma techniques, where samples can be heated because of exothermic reactions of plasma radicals on the surface of graphene, and the temperature is thus changing when plasma treatment proceeds. Therefore, it is difficult to control and keep the temperature constant during plasma synthesis.

## 4. Summary of the Literature Review

All the above-reported literature review is summarized in Table 1, Table 2 and Table 3. In Table 1 and Table 2 a summary of the methods for post-synthesis and direct- synthesis of N-doped graphene-like materials, is shown, respectively, whereas in Table 3 results of XPS characterization and treatment conditions of N-doped graphene synthesized by plasmas are summarized.

## 5. Discussion

Because of the various conditions used by authors performing plasma synthesis of N-doped graphene, such as type of the discharge, gas mixture, pressure, power, treatment time, substrate temperature, sample biased or not, etc. it is difficult to draw general correlations regarding the treatment parameters and the resulting materials’ properties. It was found that all these parameters may strongly influence the nitrogen content. Moreover, the fluxes of reactive nitrogen species from the discharge to the surface were rarely reported. Furthermore, also detailed surface chemical composition is often not reported, because the N/C ratio is often provided instead of the full composition. Many authors reported significant concentration of oxygen, for example. As shown in Table 3, the oxygen content may be very high, even exceeding the carbon content as in [37]. This points out that N-doped graphene is more susceptible to functionalization with oxygen as elaborated in [8]. Anyway, the following observations are common to the many reviewed papers:

(1) No general correlation between the nitrogen content and the characteristics of N-doped graphene samples was found. Sometimes, even very low nitrogen content of about 1 at.% was found beneficial. The induced defects increase with increasing nitrogen concentration, but this observation should be taken with a precaution because the effect of oxygen was not always included.
(2) Nitrogen is usually present in various configurations such as pyridinic, pyrrolic, and graphitic. Sometimes also oxidized nitrogen groups were reported. Usually, all three typical nitrogen configurations are found, but they differ in concentration among authors. There are only a few papers where mostly only one nitrogen configuration was reported i.e., pyridinic [34] or graphitic [36]. Therefore, it is still a challenge to control the type of nitrogen incorporated into the graphene-like structures.
(3) Treatment times for N-doping were mostly of the order of 10 min (without taking into account the time needed for the preparation of the pristine graphene samples in the case of the two-step procedure). In rare cases, treatment times of the order of 10 s were reported [29,41]. RF plasma was used in both cases.
(4) Systematic investigation of N-doping versus treatment parameters were provided in several papers. For example, the discharge power was varied systematically in [19,26], pressure in [27], and treatment time [1,11,24]. It should be stressed that such systematic experiments take time, especially spectra acquisition and the interpretation. Comparison of surface finishes obtained by different gases (N_2_ and NH_3_) using the same experimental system was reported in [29].
(5) Nitrogen content in graphene-like materials is generally higher when using N_2_-plasma than NH_3_-plasma. Furthermore, in addition to pyridinic, pyrrolic, and graphitic nitrogen, the presence of amino groups was reported for NH_3_-plasma treatments.

It is difficult to draw any clear correlation between the nitrogen concentration, its configuration, and final properties of N-doped graphene-like materials, for example its conductivity. e.g., Cho et al. found an increased conductivity of N-doped CNWs in comparison to pristine CNWs [12]. They also found that the conductivity was decreasing with increasing nitrogen content in the range of N/C from 9.5 to 22%). Different results were reported by Santhosh et al. [29], who found decreasing conductivity of N-doped CNWs for both N_2_- and NH_3_-plasma treatment, where the maximum nitrogen content was 8 and 2.8 at.% for N_2_ and NH_3_ plasma, respectively. No correlation between the measured conductivity and nitrogen content was found by Santhosh et al.

**Figure 2:**
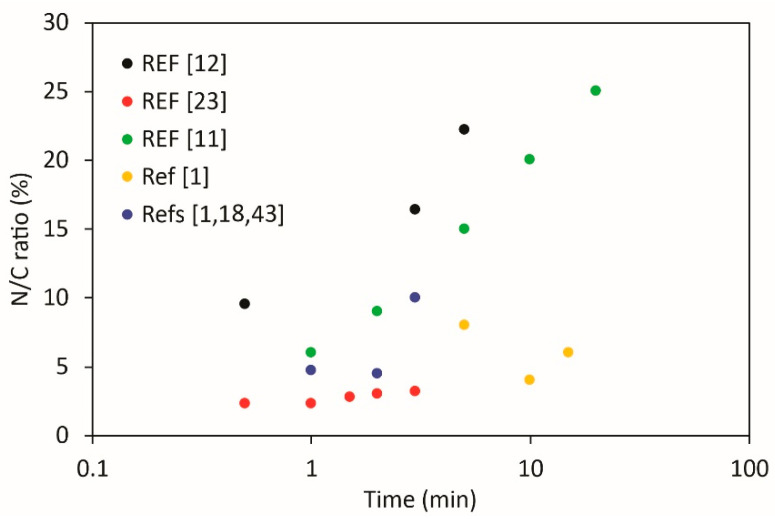
The reported N/C ratio versus the plasma treatment time.

**Figure 3:**
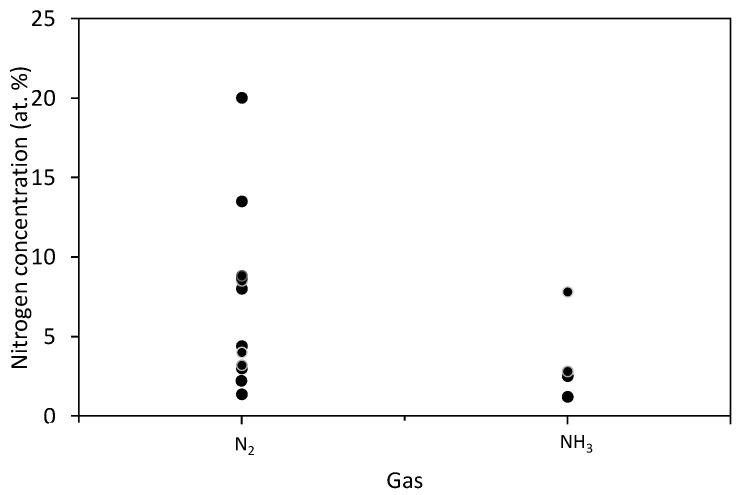
The reported nitrogen concentration versus the gas type.

**Figure 4:**
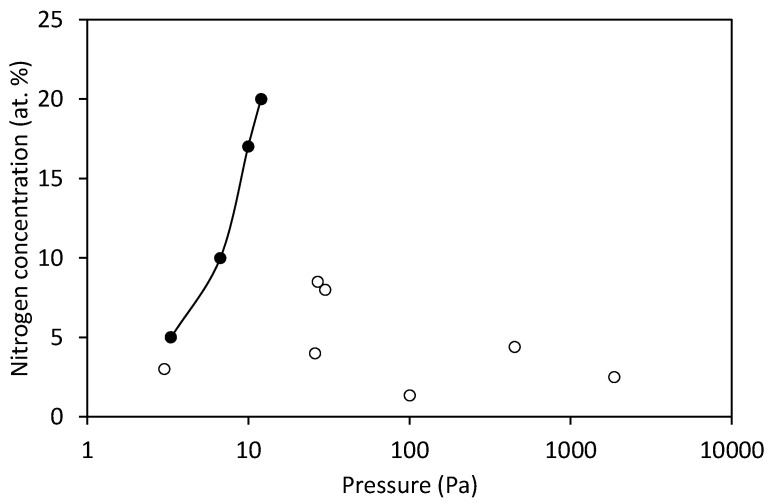
The reported nitrogen concentration versus the total gas pressure. Full symbols refer to the only more systematic study reported in [27], whereas empty symbols refer to several individual experiments of other authors mentioned in Table 3.

**Figure 5:**
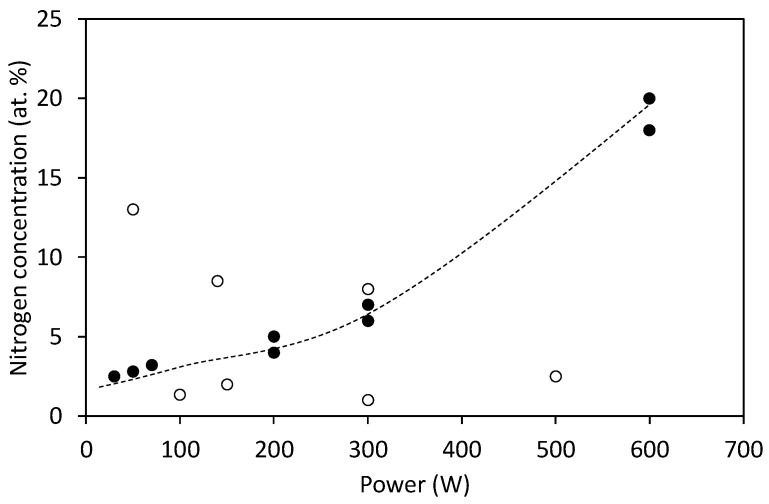
The reported nitrogen concentration versus the discharge power. Full symbols refer to two systematic investigations reported in [19,26], whereas empty symbols refer to several individual experiments of other authors mentioned in Table 3.

**Figure 6:**
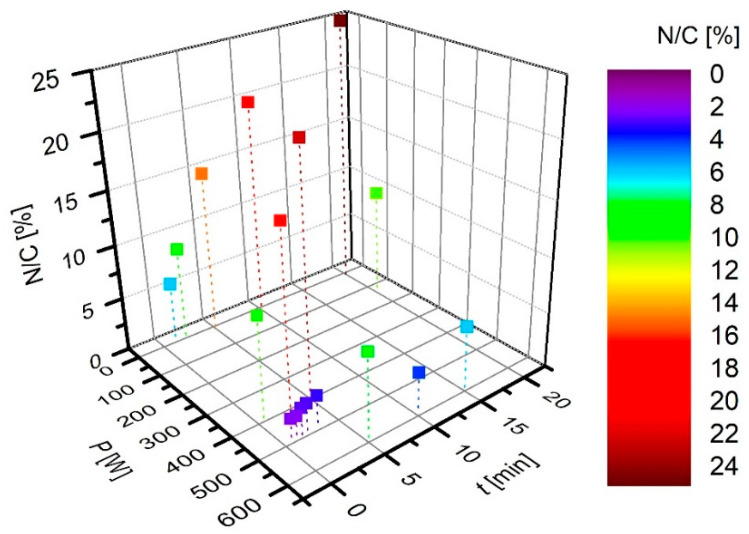
The reported N/C ratio versus two parameters, i.e., plasma treatment time and discharge power.

The results summarized in Table 3 hardly reveal any correlation between the treatment parameters and reported concentration of nitrogen in various binding sites. Despite the fact that no direct comparison is possible because of various discharge parameters, we nevertheless present the results from Table 3 graphically in Figure 2, Figure 3, Figure 4, Figure 5 and Figure 6. Many authors reported increased N-concentration with increased treatment time at their particular conditions. This is shown in Figure 2. From Figure 2 we can also see scattering of measured points obtained by between different authors, meaning that treatment time is not the only decisive parameter. When comparing applications of N_2_ or NH_3_ gas (Figure 3), it seems that more nitrogen is introduced into the graphene structure if authors used N_2_ gas. Furthermore, in the case of ammonia, authors reported the presence of amino groups [29]. There is also no clear correlation versus the gas pressure (Figure 4) and discharge power (Figure 5), although one might speculate that the N concentration is increasing with increasing power and pressure as found by some authors at their particular conditions. Nevertheless, scattering of the results can be explained as a consequence of a different concentrations and doses of reactive plasma species in the discharge operating at different powers, pressures, or treatment times. In Figure 6 is shown the reported N/C ratio versus two parameters, i.e., plasma treatment time and discharge power, but also here there is no clear correlation.

**Figure 7:**
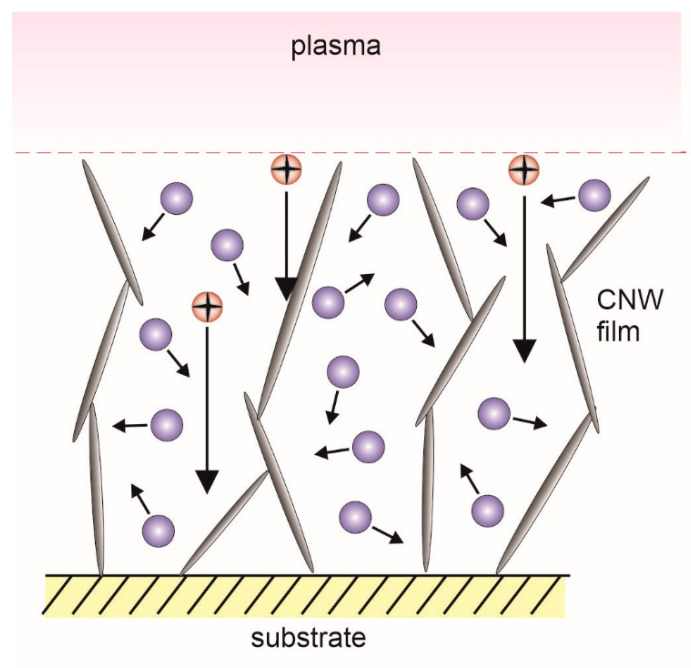
Schematic of the interaction between reactive nitrogen species and carbon nanowalls.

Finally, we should also mention the problems associated with a uniform modification of vertically-oriented graphene (CNWs). As already mentioned, such structures are typically first deposited in an atmosphere free from nitrogen, followed by a post-treatment with plasma sustained in N_2_ or NH_3_. The interaction of reactive species from nitrogen plasma with CNWs can be predicted using the schematic illustration in Figure 7. Depending on discharge parameters, there are many reactive species in nitrogen plasma, such as positively charged ions, metastable molecules, and neutral atoms. The uppermost graphene sheets are subjected to a larger flux of ions than those deep in the film. Furthermore, if the sheets are not perfectly perpendicular (very likely), the surfaces not facing plasma are not affected by ions at all. Opposite to ions, the atoms do not feel the electric field and they can move randomly, as shown schematically in Figure 7. Because the distance between the neighboring sheets is smaller than the mean free path, the collisions with surfaces will prevail. Even if the probability for recombination of N atoms is low, the huge number of collisions will create a large gradient of N-atoms across the film. In one experiment, it has been shown that the CNWs exhibit an extremely high recombination coefficient for oxygen atoms because of the porous structure [45]. The coefficient for nitrogen atoms has never been reported, but in analogy with oxygen, it can be assumed that the loss of N atoms by surface recombination to parent molecules is also large enough to create strong gradients of N atoms across the CNW film. As a result, it can be expected that CNWs deep below the surface will be under-treated.

Gradients in both positive ions and atoms are therefore likely to occur upon treatment of CNWs or similar materials with nitrogen plasma. The result is the overtreatment of the surface layer (which may lead to etching) and undertreatment of the graphene sheets deep in the structure. It is expected that the N concentration in the graphene network will be high on the surface and negligible close to the interface with the substrates. Unfortunately, we have not found a single paper on the depth profiling of CNWs to reveal uniformity of modification versus depth. Any problems with gradients are avoided if a one-step procedure is accomplished. To the best of our knowledge, no report on the growth of vertically oriented graphene structures rich in nitrogen has appeared in the scientific literature. A possible explanation is a well-known fact that any attempt to use a mixture of hydrocarbon gas and nitrogen at low pressure leads preferentially to the formation of HCN molecules rather than the deposition of nitrogenated carbon nanostructures.

## 6. Conclusions and Perspectives

N-doped graphene-like materials can be synthesized by different techniques. The most straight-forward one is chemical vapor deposition: gaseous precursors are introduced into a furnace where they gradually decompose due to high temperature and a film containing carbon and other elements grows on a substrate. This procedure will normally lead to the formation of a rather compact film, so it is used predominantly for deposition of horizontally oriented films. The technique is scalable but not very interesting for application in electrochemical devices where an extremely high surface-to-mass ratio is required. Most authors used techniques based on the application of gaseous plasma.

Gaseous plasma can be used for one-step synthesis, which is typically performed by introducing a mixture of gaseous precursors into the plasma reactor. Methane (or any other light hydrocarbon) is used as a source of carbon, and nitrogen or ammonia gas is a source of nitrogen in the graphene-like film. This technique also seems to be useful only for deposition of horizontally-oriented structures.

Three-dimensional structures of N-doped graphene-like materials of high porosity are preferably synthesized using a two-step procedure. In the first step, carbon nanowalls free from nitrogen are deposited using a PECVD technique, and these materials are then functionalized with nitrogen in the second step, where plasma free from hydrocarbons is used. The three-dimensional carbon nanomaterials are doped with nitrogen at various experimental configurations, from nitrogen or ammonia afterglows (where pure chemical interaction occurs) to biased samples where ion implantation is the predominant mechanism.

The properties of N-doped graphene-like materials vary significantly depending on their structure, which is related to the method used for their synthesis. One limitation of the methods is their ability to produce N-doped samples with controllable concentration and configuration of nitrogen, and another limitation is the upscaling of the method to mass production on the industrial scale. Two-step procedures are too time-consuming to be interesting for the industrial production of N-doped CNWs. Direct one-step procedures are thus much better alternatives. However, enabling deposition on large surface areas and line-production still remains a challenge.

The science of N-doped vertically-oriented graphene structures is still in its infancy. Numerous authors have used different plasma techniques for synthesizing such materials, which are desired in several applications, in particular the fuel cells, batteries, and supercapacitors. The obtained structures differ significantly, so no correlation between the processing parameters and the resultant structures could be drawn on the basis of reviewed literature. The concentration of nitrogen in the materials probed by XPS was from less than 1 at.% and up to about 20 at.%. Typically, it was about a few at.%. No clear correlation between the amount of nitrogen and improved characteristics of N-doped graphene samples was found. Even when nitrogen concentrations were very low at about 1 at.%, the authors reported improved characteristics of the samples. In addition, too high nitrogen concentration may again be not good enough. Several types of chemical bonds between N-atoms in the graphene structure have been confirmed. At the current level of the state-of-the-art, it is not possible to deduce the processing parameters that would lead to preferential incorporation of N-atoms to a specific binding site; therefore, this still remains a scientific challenge. The review also reveals that most authors investigated the N-incorporation in the surface film as probed by XPS and Raman spectroscopy. The results may not be representative for thick films of vertically-oriented graphene because strong gradients of fluxes of reactive nitrogen particles are expected.

The synthesis of vertically oriented N-doped graphene such as nanowalls, nanomesh, or similar morphological forms on the surface of a smooth substrate is currently limited to a two-step procedure. In the first step, a film containing nitrogen-free carbon structures is deposited, preferably by PECVD, and in the next step, the structures are exposed to nitrogen plasma. A one-step procedure currently enables deposition of horizontally oriented graphene structures of limited applicability. There is a need for inventing a method for a one-step deposition because the gradients in nitrogen concentration could be suppressed if not avoided using such a technique. Any attempt to deposit vertically oriented N-doped carbon nanostructures using hydrogenated carbon precursors in plasma with nitrogen or ammonia admixtures failed, probably due to preferential etching and formation of volatile hydrogen cyanide.

The deposition of vertically oriented carbon nanostructures with an appropriate concentration of nitrogen in various chemical bonds remains a scientific challenge. In order to enlighten the kinetics of the formation of various nitrogen configurations in graphene structure, the correlations between the fluxes and/or fluences of reactive nitrogen plasma particles and surface finish are desired. Once the correlations are known, further studies will be necessary to form desired structures in a controllable and repeatable manner.

## Figures and Tables

**Table 1:** Overview of published literature on post-synthesis of N-doped graphene.

Ref	Method	Gas	Material	Treatment Parameters for N-Doping	Methods for Characterization	Most Important Conclusions	Possible Application
[1]	Post-plasma treatment	N_2_, O_2_ or mixture O_2_:N_2_ = 25:75	CNWs	DC discharge, 2kV, 80 mA, pressure: 0.2 Torr, treatment time: 1–120 min (90 min)	SEM, XPS, Raman (/), CV	- 4 at.% N + 10 at.% O, - increased specific capacitance - *I*_D_/*I*_G_ increased from 0.81 to 0.86,1.63, and 1.38 for O_2_, N_2_, and O_2_/N_2_, respectively; *- I*_2D_/*I*_G_ decreased from 0.95 to 0.72, 0.32, and 0.64 for O_2_, N_2_, and O_2_/N_2_, respectively, *- L*_a_ decreased from 23.8 nm to 22.4, 11.8, and 14 nm for O_2_, N_2_, and O_2_/N_2_, respectively	supercapacitors
[18]	Post-plasma treatment	N_2_	CNWs	DC discharge, pressure: 3 Pa, treatment time: 2 h	SEM, XPS, Raman (/), electrochemical measurements	3 at.% N + 30 at.% O *I*_D_/*I*_G_ increased from 0.78 to 0.90 after doping	supercapacitors
[12]	Post-plasma treatment	N_2_	CNWs	CCP plasma, power: 400 W, N_2_ flow 10 sccm, treatment time: 30–300 s	OES, SEM, XPS, Raman (/), van der Pauw-Hall measurements	Electrical properties depended on treatment time. N/C = 9.5% (30 s)–22.2% (300 s)	electronic application
[19]	Post-plasma treatment	N_2_/Ar(1:2)	CNWs	Pressure: 2 Pa, RF power: 200, 300, 600 W, treatment time: 15 min	XPS, Raman (632.8 nm), TEM, RDE	4–20 at.% of N 39–52 at.% of O, A change of *I*_D_/*I*_G_ after doping depended on the crystallite size *L*_a_ of non-doped CNWs.	Electrocatalyst for polymer electrolyte membrane fuel cells
[20]	Post-plasma treatment	NH_3_	Vertical graphene nanowalls	MW PECVD, In-situ doping after CNW synthesis using NH_3_	SEM, TEM, XPS, Raman (532 nm), EIS, CV, galvanostatic measurements	7.8 at.% of N—only pyridinic N was found. Capacitance: 991.6 F/g, Energy density: 275.4 Wh/kg, Power density: 14.8 kW/kg	supercapacitors
[30]	Ion implantation	N_2_	Vertical graphene nanowalls	RF source of ions, Sample biased with pulsed DC voltage of 2 kV; Treatment time: 10, 20, 30 min	SEM, Raman (514.5 nm), XPS, AFM/AFAM	7.6–8.8 at.% of N and ~13 at.% of O. Reduction of *I*_D_/*I*_G_ from 2.5 to 1.3. No modification in CNWs morphology up to 20 min.	/
[21]	Post-plasma treatment	NH_3_	Vertically aligned few-layer graphene (FLG)	PECVD, RF power: 20 W, pressure: 1.4 Torr, NH_3_ flow rate 50 sccm, substrate temperature: 350 °C, treatment time: 30 min	SEM, TEM, XPS, Raman (514.5 nm), field emission properties	1.2 at.% of N in the form of amino groups. *I*_G_/*I*_2D_ decreased from 1.53 to 1.03, whereas *I*_D_/*I*_G_ increased from 1.94 to 2.20. Lower work function and enhanced electron emission properties.	Field emitters
[22]	Post*-plasma treatment *already pristine CNW contaminated with N	Ar/N_2_ or Ar/O_2_	CNWs	PECVD, RF power 50 W Ar flow 100 sccm, N_2_ or O_2_ flow 10 sccm, pressure: 0.2 Pa, treatment time: 5 min	SEM, XPS, Raman (514 nm), CV, EIS	12.5–13.5 at.% of N. Pyrrolic N was found to be important for improvement of electrochemical transaction. *I*_2D_/*I*_G_ decreased from 0.5 to 0.4 and 0.2, whereas *I*_D_/*I*_G_ decreased from 1.47 to 1.38 and 1.27 for Ar/N_2_ and Ar/O_2_, respectively	Electrochemical transductors
[23]	Post-plasma treatment	N_2_	Graphene layer	PECVD, power: 500 W, pressure: 14 Torr, flow rate 91 sccm, treatment time: up to 3 min followed by annealing at 300 °C for 3 h	TEM, SAED, XRD, SPEM, Raman (/), CV, galvanostatic measurements	1.7–2.5 at.% of N, 16–25 at.% of O, capacitance 4× larger than for the undoped graphene (280 F/g_electrode_), excellent cycle life	ultracapacitors
[11]	Post-plasma treatment	NH_3_	Graphene oxide monolayer	DC plasma, power: 10 W, pressure: 1 Pa, treatment time 1–20 min	SEM, AFM, XPS, UPS, Raman (514 nm), electrical conductivity	N/C = 6–25%, O/C = 15–27%. Pyridinic, pyrrolic, and graphitic N content depended on treatment time. The best results obtained at low treatment time (n-type). *I*_D_/*I*_G_ increased from 1.5 to 1.9 only for long times.	/
[24]	Post-plasma treatment	Ar followed by NH_3_/H_2_	Graphene	MW, remote two-step procedure: Ar plasma (60 s), followed by NH_3_/H_2_ (300 s), Ar flow = 200 sccm (2 Torr), NH_3_ flow = H_2_ flow = 50 sccm (1 Torr), sample position 30 cm downstream	XPS, Raman (532 nm), electrical measurements	2.5 at.% of N, n-type *I*_2D_/*I*_G_ = 1.2, *I*_D_/*I*_G_ = 0.02 for pristine graphene. After Ar treatment *I*_D_/*I*_G ~_ 2.5, after Ar/NH_3_/H_2_ treatment *I*_D_/*I*_G ~_ 1	transistors
[37]	Post-plasma treatment	Ar/N_2_	Self-standing graphene sheets	MW power 600 W, Pressure: 100 Pa, total flow 50 sccm, N_2_:Ar = 10:90, treatment time: 5, 10, and 15 min	Raman (633 nm), XPS, TEM, OES	Pyridinic, pyrrolic and quaternary N, high doping level 5.6%, increase of sp^2^/sp^3^ ratio	/
[38]	Post-plasma treatment	NH_3_	Bilayer graphene	Dose: 3 × 10^14^ cm^−2^, other details not specified	XPS, Raman (633 nm)	Doping level: 1.5 × 10^13^ cm^−2^. *I*_2D_/*I*_G_ changed from 1.7 to 0.7	/
[39]	Post-plasma treatment	NH_3_	Graphene sheet	RF 13.56 MHz, with/without an additional Cu grid in the discharge tube after the coil. Power 20 (45 W) with (without) a grid. Remote treatment at a distance 75 (45) cm and treatment time 60 (10) min with (without) a grid.	AFM, Raman (632.8 nm), electrical measurements	Graphene preferably doped near the edge. Doping density: 1.7 × 10^12^ cm^−2^ for mild treatment (with a grid).	/
[25]	Post-plasma treatment	N_2_	Graphene monolayer	Tunable hybrid ECR-MW plasma source, two modes of operation: (1) an ion-mode with a flux: 4 × 10^12^ ions s^−1^ cm^−2^, energy 35 eV, and (2) an atom-mode (by using an ion trap) with a flux of atoms 2.5 × 10^15^ s^−1^ cm^−2^, sample at 850 °C, pressure 5 × 10^−5^ mbar, treatment time: 10 min	ARIPES, XPS, LEED	Ion-mode treatment: n-type dopping attributed to 8.7 at.% of graphitic N. Atom-mode treatment:mainly pyridinic N is formed, minor n-doping	/
[40]	Post-plasma treatment	N_2_	Few-layer graphene	Ion irradiation, DC power supply, negative bias 300–350 V, pressure: 460 Pa, treatment time: 20 and 40 s	XPS, Raman (532 nm), TEM, EIS	4.4 and 2.8 at.% of N for 40 and 20 s. Mostly pyridinic and pyrrolic N, graphitic only in a minor concentration. 3-times higher energy conversion efficiency.	Solar cells
[28]	Post-plasma treatment	N_2_	Mono-, few-, and multi-layer graphene	APPJ (15 kV, 25 kHz, AC), flow rate 15 slm, APPJ positioned in N_2_ surrounding atmosphere, treatment times: 1–30 s, jet distances from the sample: 1, 2, 3 cm	XPS, TEM, WCA, Raman (532 nm)	Pyridinic nitrogen prevailed. *I*_D_/*I*_G_ increased with plasma treatment time from 0.22 to 0.6. Surface change to hydrophilic (contact angle 44°) because of the OH and COOH groups.	/
[41]	Post-plasma treatment	N_2_	Monolayer graphene	RF plasma 13.56 MHz, power: 10 W, pressure: 0.12 Torr, treatment time: 0–16 s	XPS, Raman (514.5 nm), CV, RDE	Pyridinic N prevailed, followed by pyrrolic and graphitic N. Enhanced electrocatalytic activity and charge transfer.	Hydrogen production
[42]	Post-plasma treatment	N_2_	Graphene	Harrick model PDC-32G plasma cleaning unit, power: 100 W, pressure: 0.75 Torr, treatment time: 20, 40, 60, 100 min	XPS, TEM, CV,	1.35 at.% of N and 28 at.% of O. High electrochemical activity for reduction of H_2_O_2_. Fast direct electron transfer kinetics for glucose oxidase	Biosensors
[43]	Post-plasma treatment	N_2_	Graphene sheet	PC2000—Plasma Cleaner, RF 13.56 MHz, power 140 W, pressure: 0.2 Torr, treatment time: 20 min, DC bias 990 V	XPS, Raman (514.5 nm), ORR, CV	8.5 at.% of N and 8.6 at.% of O. Nitrogen was in all typical configurations with the highest pyrrolic content. Higher electrochemical activity toward oxygen reduction	ORR (fuel cells, biosensors)
[26]	Post-plasma treatment	N_2_	Graphene films	RF, powers: 30, 50, 70 W, flow rate: 50 sccm, pressure: 0.7 Pa, treatment time: 5 min	XPS, Raman (532 nm), Scanning Kelvin Probe, Van der Pauw-Hall measurements	n-type, mostly graphitic N. *I*_D_/*I*_G_ increased from 0.42 to 0.45, 0.60, 0.81 for 30, 50, and 70 W, respectively. Increased power caused increased graphitic content, increased electron concentration, and a shift of Fermi level to higher energy. Work function decreased.	optoelectronics
[27]	Post-plasma treatment	Ar/NH_3_	Graphene films	Electron beam plasma, 2 kV, 5% NH_3_, pressure: 25–90 mTorr, total treatment time: 60 s (equivalent plasma exposure time 6 s)	XPS, Raman (/)	N content increased with increasing pressure from 5 to 20 at.%. Raman D peak also increased with pressure.	biosensors
[29]	Post-plasma treatment	N_2_ or NH_3_	Graphene nanowalls	IC RF, power: 300 W, flow rate: 100 sccm, pressure: 30 Pa, post-glow region i.e., 10 cm away from the glow, treatment time: 4, 8, 12, 25 s (for NH_3_) and 10, 20, 30, 40 s (for N_2_), pulsed treatment to keep the sample < 50 °C	XPS, SEM, NEXAFS, Raman (633 nm), van der Pauw measurements	8.0 and 2.8 at.% of N for N_2_ and NH_3_, respectively. All three N types were found as well as amine for NH_3_ treatment. N_2_ caused etching, which was not observed for NH_3_. *I*_D_/*I*_G_ was in general decreasing with increasing treatment time: from 2.8 to 2.28 (40 s, N_2_) or to 2.68 (12 s, NH_3_).	/

**Table 2:** Overview of published literature on direct-synthesis of N-doped graphene.

Ref	Method	Gas/Precursor	Material	Treatment Parameters for N-Doping	Methods for Characterization	Most Important Conclusions	Possible Application
[9]	CVD	s-triazine	Graphene monolayer	vapor pressure: 1 × 10^−6^ mbar, deposition time: 30 min, temperature: 540–635 °C.	XPS, ARPES, NEXAFS	1–2 at.% of N (0.4 at.% of graphitic N) Bandgap 0.2 eV	semiconductors
[14]	Direct plasma synthesis	Ethanol + NH_3_	Free-standing graphene	MW at atmospheric pressure, additional IR and UV treatment for sp^2^ C and N-type manipulation. Deposition yield: 1.3 mg/min	XPS, SEM, TEM, Raman (633 nm), CV, van der Pauw method, OES and FTIR	N/C = 0.4%,O/C = 1.5%*I*_D_/*I*_G_ ~ 0.9 after irradiation; Higher relative amount of pyridinic and pyrrolic N for the irradiated CNWs.	supercapacitors
[44]	Thermal segregation		Few-Layer graphene	Annealing of a substrate consisting of N-containing boron and C-containing Ni films	XPS, Raman (514.5 nm), AFM, electrical characteristic of fabricated field-effect transistors	Higher N doping caused lower *L*_a_. *L*_a_ reduced from 65 nm to 21 and 8 nm for N/C = 0.6 and 2.9%, respectively Doping level 4 × 10^13^ cm^−2^, bandgap 0.16 eV, n-type	nanoelectronics
[31]	Chemical synthesis	CCl_4_ + Li_3_N	Few-layer graphene	Reaction of CCl_4_ with Li_3_N	STM, TEM, XPS, Raman (633 nm), thermal stability tests	N/C = 4.5–16.4%. In the sample with a high N content, pyridinic and pyrrolic N dominated (p-type). For the sample with a low N content, graphitic N dominated (n-type).	nanoelectronics
[32]	CVD	1,3,5-triazine	Graphene sheets	Chemical vapor deposition of 1,3,5-triazine to Cu substrate at different temperatures 700, 800 and 900 °C	XPS, Raman (473 nm), AFM, SEM, TEM, electrical measurements	N/C = 2.1–5.6%. A lwer temperature was favorable to obtain higher N doping. Increasing of N-doping content caused the transformation of p-type to n-type.	nanoelectronics
[4]	CVD	CH_4_ + NH_3_	Few-layer graphene	NH_3_/CH_4_/H_2_/Ar = 10/50/65/200 sccm for 5 min, followed by NH_3_/Ar for another 5 min, temperature 1000 °C	AFM, TEM, Raman (514.5 nm), XPS, SEM, XRD, RDE	4 at.% of N, pyridinic, and pyrrolic N-configuration. *I*_D_/*I*_G_ = 0.06–0.25 Improved electrocatalytic activity and stability.	Fuel cells
[33]	In-liquid plasma	ethanol + Fe-phthalocy-anine	Nano-graphenes	In-liquid plasma synthesis from ethanol and Fe-phthalocyanine	SEM, XPS, Raman (/), ORR, CV	6–11 at.% of N, N-configurations: pyridinic, Fe-N, pyrrolic and graphitic. *I*_D_/*I*_G_ = 1.25–1.66, *L*_a_ = 11.6–15. 3 nm	Polymer electrolyte fuel cells
[16]	PECVD	H_2_/CH_4_/N_2_	Mono- to multilayer graphene	Flow rates of H_2_/CH_4_/N_2_ = 20/5/1 sccm. Power 300 W, pressure 1.08 Pa, growth time: 5 min, annealing to 500 and 950 °C.	XPS, Raman (532 nm)	N content: 0.5, and 1.1% at 950 and 500 °C, respectively. N mostly in the graphitic form. *I*_2D_/*I*_G_ = 2.1 (decreasing with N content). *I*_D_/*I*_G_ = 1–1.5. An island like growth.	/
[17]	PECVD	H_2_/CH_4_/N_2_	Few-layer graphene	MW, first H_2_/CH_4_ treatment at 500 W, followed by N_2_/CH_4_ treatment at 150 W. Pressure: 10 Torr, flow: H_2_ = CH_4_ = 10 sccm, N_2_ = 50 sccm. Total growth time: 5 min. Temperature: 800 °C.	Raman (532 nm), XPS, SEM, TEM	2 at.% of N in the form of pyridinic, graphitic, and oxygenated form. *I*_D_/*I*_G_ increased from 1.34 to 2.3, and *I*_2D_/*I*_G_ decreased from 1.0 to 0.28.	/
[15]	PECVD	Ar/ethanol/N_2_ +UV	Free- standing graphene	MW, power: 2 kW, Ar flow 1200 sccm, ethanol flow 15 sccm, N_2_ flow 5 or 10 sccm	XPS, SEM, FTIR, NEXAFS, Raman (532 nm)	0.2 at.% of N and 8 at.% of O, mostly pyridinic nitrogen and some graphitic, growth yield 2 mg/min.	/
[8]	PECVD	H_2_/CH_4_/N_2_	Graphene bilayers	MW, power: 500 W, N_2_:CH_4_ = 2:1, 3:1, or 5:1, H_2_ flow: 10 sccm, pressure: 43 Torr, deposition time: 2.5 min, temperature: 760 °C	XPS, Raman (532 nm), simulations	2.0–4.2 at.% of N, pyridinic, and another peak related to other type of N defects. Formation of interlayer bonds mediated by nitrogen defects. *I*_D_/*I*_G_ increased from 0.6 to 2, *I*_2D_/*I*_G_ decreased from 1.5 to 0.7.	/
[34]	CVD	H_2_/C_2_H_4_/ NH_3_	Single-layer graphene	Thermal deposition in H_2_/C_2_H_4_/NH_3_ at various NH_3_ flow rates	XPS, Raman (532 nm), SIMS, UPS, RDE voltametry	N/C = 1.6–16%. Depending on NH_3_ flow, pure pyridinic N formation.	ORR
[35]	CVD	CH_4_/NH_3_	Graphene domain film	Thermal deposition in NH_3_ and CH_4_ at various temperatures 880–1050 °C	XPS, Raman (532 nm)	Control of N configuration by growth temperature. At high temperatures, mostly pyridinic N was formed, and pyrrolic N at low temperatures. The N concentration was decreasing with increasing temperature. N = 4.5 and 0.7 at.% at 880 and 1050 °C, respectively.	/
[13]	Free-radical reaction	Penta-chloro-pyridine	Graphene films	Free-radical reaction from pentachloropyridine at various growth temperatures 230–600 °C	XPS, Raman, STM, electronic properties	Control of N configuration by growth temperature. Graphitic N dominated at 230–300 °C and pyrrolic N at (400–600 °C). *I*_D_/*I*_G_ = 0.48–1.91 (minimum at 400 °C) *L*_a_ = 7.4–19.6 nm	/

**Table 3:** Summary of discharge parameters and XPS results for the direct and post-plasma synthesis of N-doped graphene.

Ref	Gas	Discharge	Material	Treatment Time	Power	Pressure/Flow	N and O Content as Obtained by XPS
[1]	N_2_, O_2_, or mixture	DC	CNWs	1–120 min (90 min)		0.2 Torr	N: 4 at.% O: 10 at.% N/C = 4.7%
[18]	N_2_	DC	CNWs	2 h		3 Pa	N: 3 at.% O: 30 at.% N/C = 4.5%
[12]	N_2_	CCP	CNWs	30, 180, 300 s	400 W	10 sccm	N/C = 9.5% at 30 s N/C = 16.4% at 180 s N/C = 22.2% at 300 s
[19]	N_2_/Ar	RF	CNWs	15 min	200, 300, and 600 W	2 Pa	N: 5, 7, or 18 at.%, O: 41, 52 or 39 at.%, N/C = 9, 17 or 42% for 200, 300 and 600 W, respectively (pristine CNW deposited at 860 °C). N: 4, 6, or 20 at.% O: 47, 45, 39 at.% N/C = 8, 12 or 49% for 200, 300 and 600 W, respectively (pristine CNW deposited at 730 °C)
[20]	NH_3_	MW PECVD	Vertical graphene nanowalls				N: 7.8 at.%, pyridinic N
[30]	N_2_	RF/DC biased	Vertical graphene nanowalls	10, 20, 30 min			N: 7.6–8.8 at.% O: ~13 at.% N/C ~ 10%
[21]	NH_3_	RF PECVD	Vertically aligned few-layer graphene (FLG)	30 min	20 W	1.4 Torr 50 sccm	N: 1.2 at.%, amino groups
[22]	Ar/N_2_ or Ar/O_2_	RF PECVD	CNWs	5 min	50 W	0.2 Pa 100/10 sccm	N: 12.5–13.5 at.% Pyridinic, pyrrolic, graphitic, and oxygenated N
[23]	N_2_	PECVD	Graphene layer	0.5–3 min + followed by annealing 3 h	500 W	14 Torr 91 sccm	N: 1.7 at.%, O: 25.5 at.%, N/C = 2.3% for 0.5 min N: 1.9 at.%, O: 15.9 at.%, N/C = 2.3% for 1 min N: 2.2 at.%, O: 21.8 at.%, N/C = 2.8% for 1.5 min N: 2.4 at.%, O: 16.9 at.%, N/C = 3.0% for 2 min N: 2.5 at.%, O: 19.6 at.%, N/C = 3.2% for 3 min Pyridinic, pyrrolic and graphitic. Graphitic content was decreasing with increasing treatment time.
[11]	NH_3_	DC	Graphene oxide monolayer	1–20 min	10 W	1 Pa	N/C = 6%, O/C = 27%, for 1 min N/C = 9%, O/C = 25% for 2 min N/C = 15%, O/C = 15% for 5 min N/C = 20%, O/C = 22% for 10 min N/C = 25%, O/C = 26% for 20 min Pyridinic, pyrrolic and graphitic
[24]	Ar + NH_3_/H_2_	MW	Graphene	60 s in Ar + 300 s in NH_3_/H_2_		1 Torr, 200/50/50 sccm	N: 2.5 at.%
[37]	Ar/N_2_	MW	Self-standing graphene sheets	5, 10, 15 min	600 W	100 Pa 45/5 sccm	N/C ~ 8%, O/C ~ 19% for 5 min N/C ~ 4%, O/C ~ 26% for 10 min N/C ~ 6%, O/C ~ 115% for 15 min (estimated from the graph) Pyridinic, pyrrolic, and graphitic N
[25]	N_2_	ECR-MW	Graphene monolayer	Two modes of operation: (1) ion-mode, flux = 4 × 10^12^ ions s^−1^ cm^−2^, (2) atom-mode, flux = 2.5 × 10^15^ s^−1^ cm^−2^,		0.005 Pa	Ion-mode treatment: N: 8.7 at.%, mostly graphitic Atom-mode treatment: Minor doping, mainly pyridinic
[40]	N_2_	DC biased	Few–layer graphene	20 s, 40 s		460 Pa	N: 4.4 at.% at 40 s N: 2.8 at.% at 20 s Mostly pyridinic and pyrrolic, graphitic N in a minor concentration
[41]	N_2_	RF	Monolayer graphene	14 s	10 W	0.12 Torr	2.2 at.%, pyridinic prevails, followed by pyrrolic and graphitic
[42]	N_2_	Harrick model PDC-32G plasma cleaning unit	Graphene	20, 40, 60, 100 min	100 W	0.75 Torr	N: 1.35 at.% O: 28 at.% N/C = 1.9%
[43]	N_2_	RF biased (PC2000—Plasma Cleaner)	Graphene sheets	20 min	140 W	0.2 Torr	N: 8.5 at.% O: 8.6 at.% N/C = 10% Pyridinic, pyrrolic (the highest content), and graphitic
[26]	N_2_	RF	Graphene films	5 min	30, 50, 70 W	0.7 Pa	N: 2.5, 2.8 and 3.2 at.%, for 30, 50 and 70 W, respectively Mostly graphitic
[27]	Ar/NH_3_	Electron beam plasma	Graphene films	total treatment time 60 s (equivalent plasma exposure time 6 s)		25–90 mTorr	N: 5 at.% at 3.3 Pa N: 10 at.% at 6.7 Pa N: 17 at.% at 10 Pa N: 20 at.% at 12 Pa
[29]	N_2_ or NH_3_	RF	Graphene nanowalls	4, 8, 12, 25 s (for NH_3_) 10, 20, 30, 40 s (for N_2_)	300 W	30 Pa	N: 8.0 and 2.8 at.% for N_2_ and NH_3_, respectively Pyridinic, pyrrolic and graphitic N, as well as amine in the case of NH_3_ treatment
[14]	EtOH/ NH_3_	MW	Free-standing graphene	/		Atmospheric	N/C = 0.4%, O/C = 1.5% Higher amounts of pyridinic and pyrrolic N, if irradiated.
[33]	EtOH and Fe-phthalo-cyanine	In-liquid plasma	Nano-graphenes				N: 6–11 at.% Pyridinic, Fe-N, pyrrolic, and graphitic N
[16]	H_2_/CH_4_/N_2_	PECVD	Mono- to multilayer graphene	5 min	300 W	1.08 Pa	N: 0.5%–1.1% for 950 and 500 °C, respectively Mostly graphitic N.
[17]	H_2_/CH_4_/N_2_	PECVD	Few-layer graphene	5 min	500 W	10 Torr 10/10/50 sccm	N: 2 at.% Pyridinic, graphitic and oxygenated N
[8]	N_2_/H_2_/CH_4_	MW PECVD	Graphene bilayers	2.5 min	500 W	43 Torr	N: 2.0 at.% for N_2_:CH_4_ = 2:1 N: 4.2 at.% for N_2_:CH_4_ = 3:1 and 5:1 Pyridinic N and another one related to other type of defects
[15]	Ar/EtOH/N_2_	MW PECVD + UV	Free- standing graphene	/	2000 W	1200/15/(5 or 10) sccm	N: 0.2 at.% O: 8 at.% Mostly pyridinic and some graphitic

**Figure A1:**
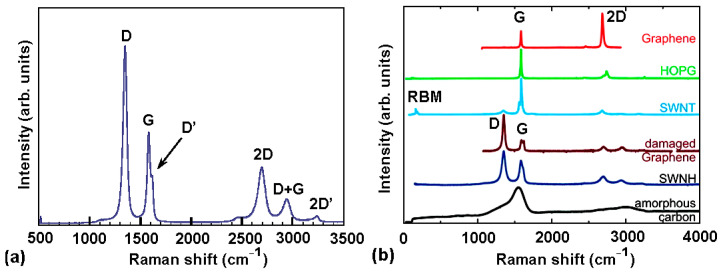
(**a**) Raman spectra of CNWs (Reproduced from [47] (Hiramatsu et al.) under CC Attribution 3.0), and (**b**) comparison of Raman spectra of various types of sp^2^ nanocarbon configurations such as graphene, HOPG (highly-oriented pyrolytic graphite), SWNT (single-walled nanotube), SWNH (Single-walled nanohorn), and amorphous sp^3^ carbon. Reprinted with permission from Dresselhaus et al. [48] Copyright 2020 American Chemical Society.

**Figure A2:**
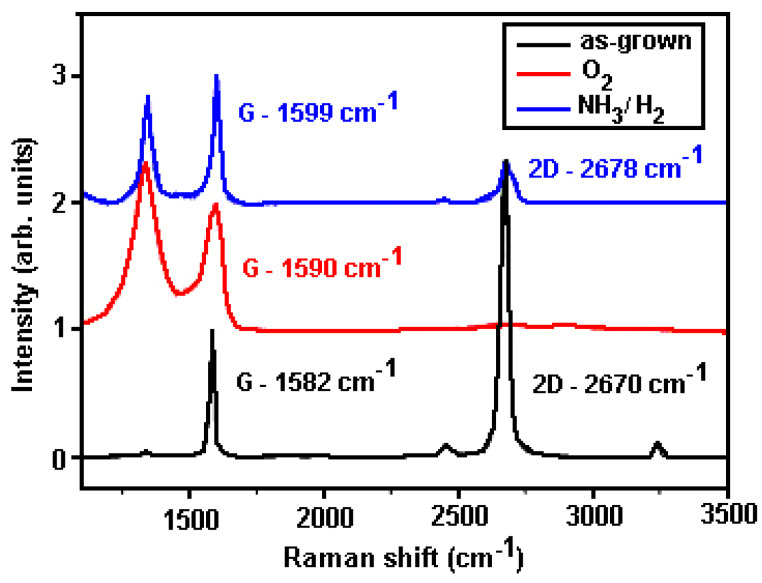
Raman spectra of pristine graphene, graphene treated with O_2_ plasma, and O_2_ plasma-treated graphene after additional treatment in NH_3_/H_2_ plasma. Reprinted from McEvoy et al. [51]. Copyright 2020, with permission from Elsevier.

**Figure A3:**
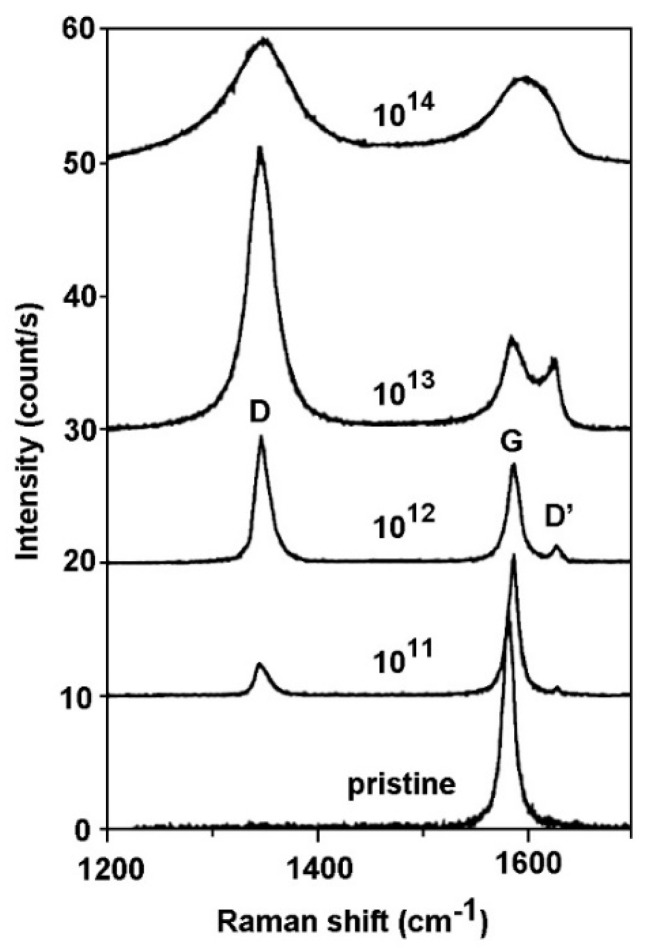
Evolution of Raman spectra of graphene samples exposed to Ar ions. The ions doses are in units of cm^−2^. Reprinted from Lucchese et al. [52]. Copyright 2020, with permission from Elsevier.

**Figure A4:**
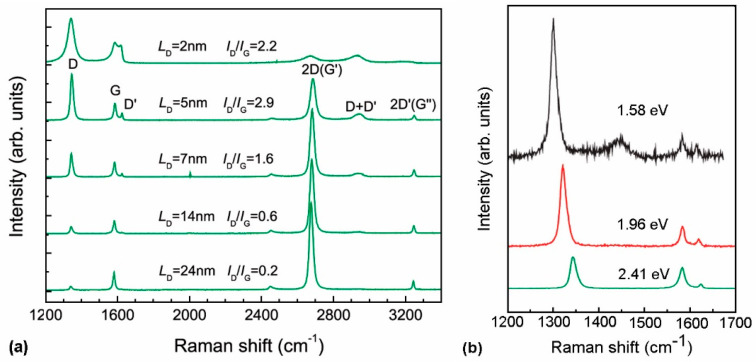
(**a**) Raman spectra of ion-bombarded single-layered graphene samples with different interdefect distance, and (**b**) Raman spectra of ion-bombarded single-layered graphene measured at different excitation laser wavelengths (laser energies). Reprinted with permission from [53] (Conçado et al.) Copyright (2020) American Chemical Society.

**Figure A5:**
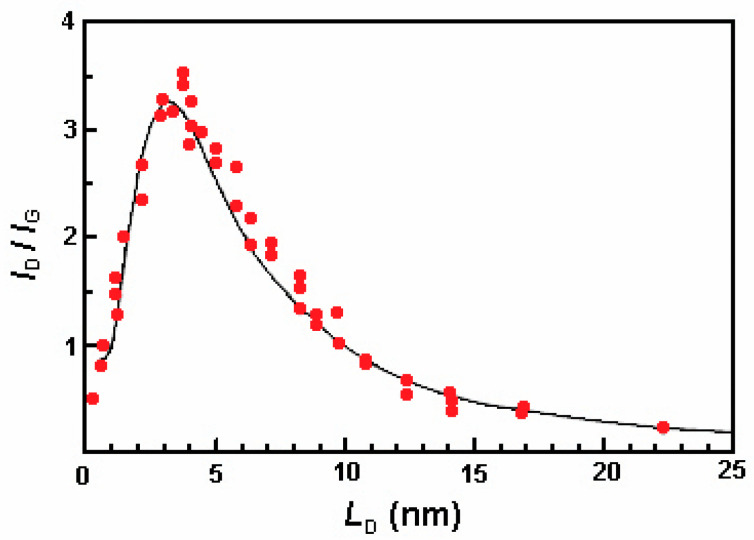
The *I*_D_/*I*_G_ ratio of mono-layered graphene samples (using a *λ* = 514 nm laser) versus the distance between defects induced by ion bombardment. Reprinted from Lucchese et al. [52]. Copyright 2020, with permission from Elsevier.

**Figure A6:**
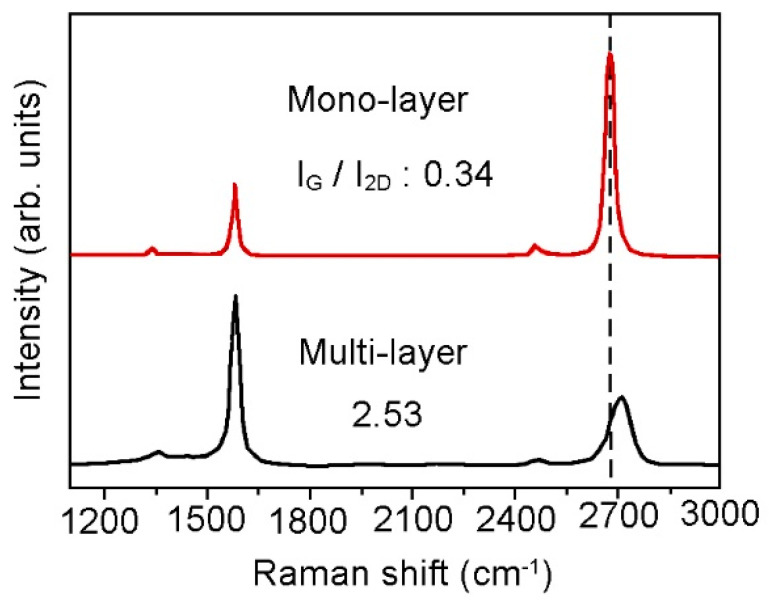
An example of the Raman spectrum of mono- and multi-layer graphene showing different 2D to G ratio. Reprinted from Lee et al. [28]. Copyright 2020, with permission from Elsevier.

**Figure A7:**
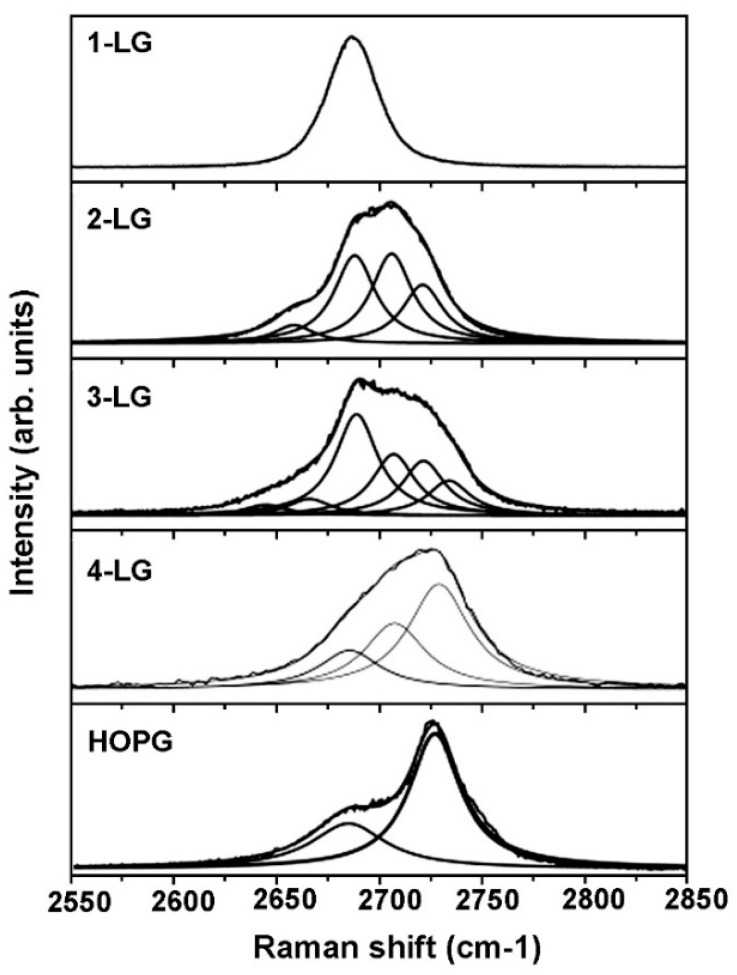
Changes in the shape of the 2D band measured for single, double, triple, and four-layered graphene samples. Reprinted from Malard et al. [59]. Copyright 2020, with permission from Elsevier.

**Figure A8:**
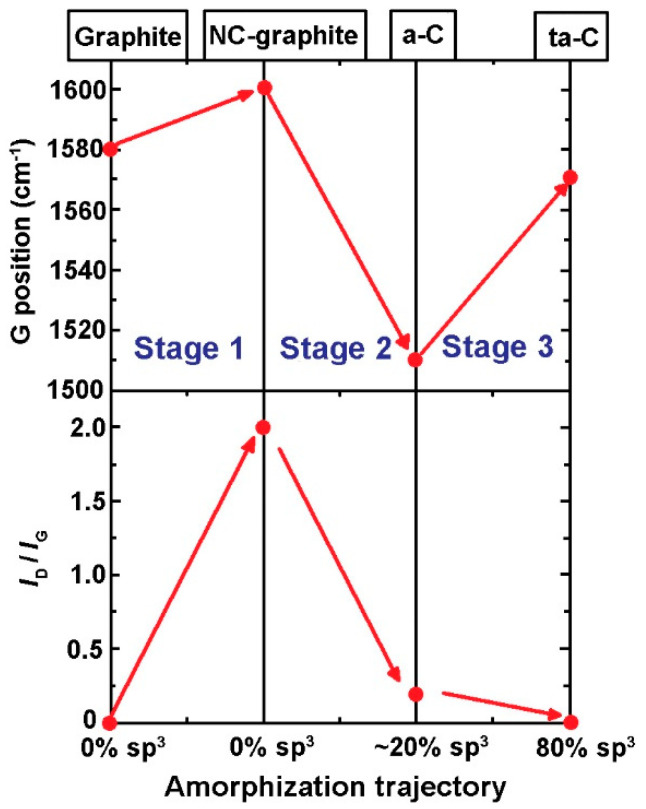
Amorphization trajectory showing the dependence of G-band position and *I*_D_/*I*_G_ for various sp^3^ contents during the transition between different stages. Reprinted with permission from Ferrari et al. [50]. Copyright (2020) by the American Physical Society.

**Figure A9:**
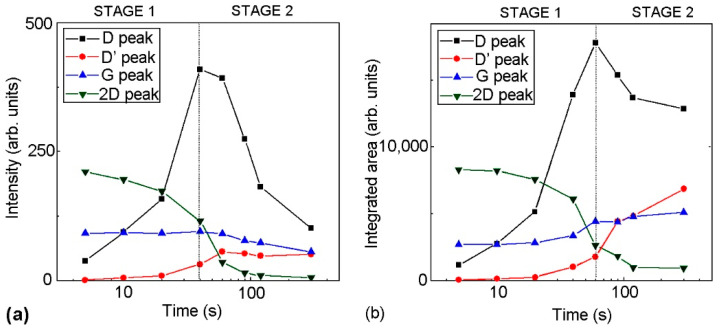
Difference between maximum and integrated peak intensities-variation of Raman intensities of characteristic bands versus plasma treatment time, showing the transition from stage 1 to stage 2: (**a**) maximum peak intensities, and (**b**) integrated peak areas. We can observe that in stage 2, there is a difference in the behavior of maximum intensities or integrated areas, especially of the G and D peaks. Reprinted with permission from [60] (Eckmann et al.). Copyright (2020) American Chemical Society.

**Figure A10:**
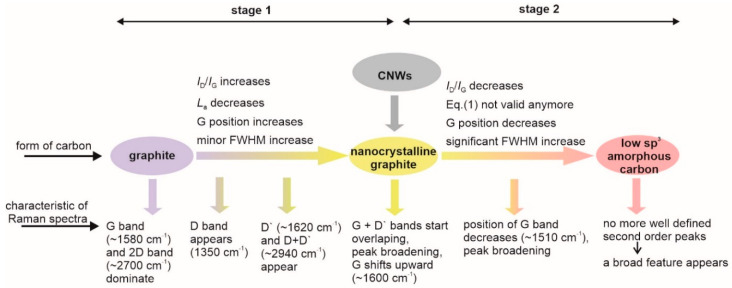
The most important steps in the evolution of Raman spectra for the case, when graphite is changed to nanocrystalline graphite (stage 1) or from nanocrystalline graphite to low sp^3^ amorphous carbon (stage 2).

**Figure A11:**
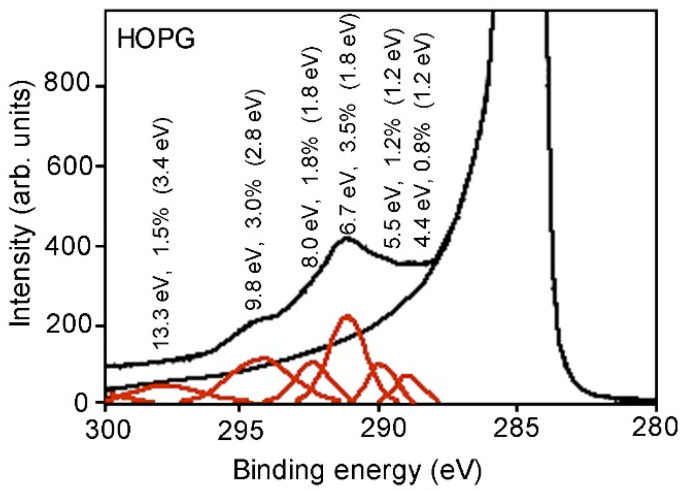
Position of satellites, its relative intensity and FWHM for HOPG. Reprinted from Leiro et al. [61]. Copyright 2020, with permission from Elsevier.

**Figure A12:**
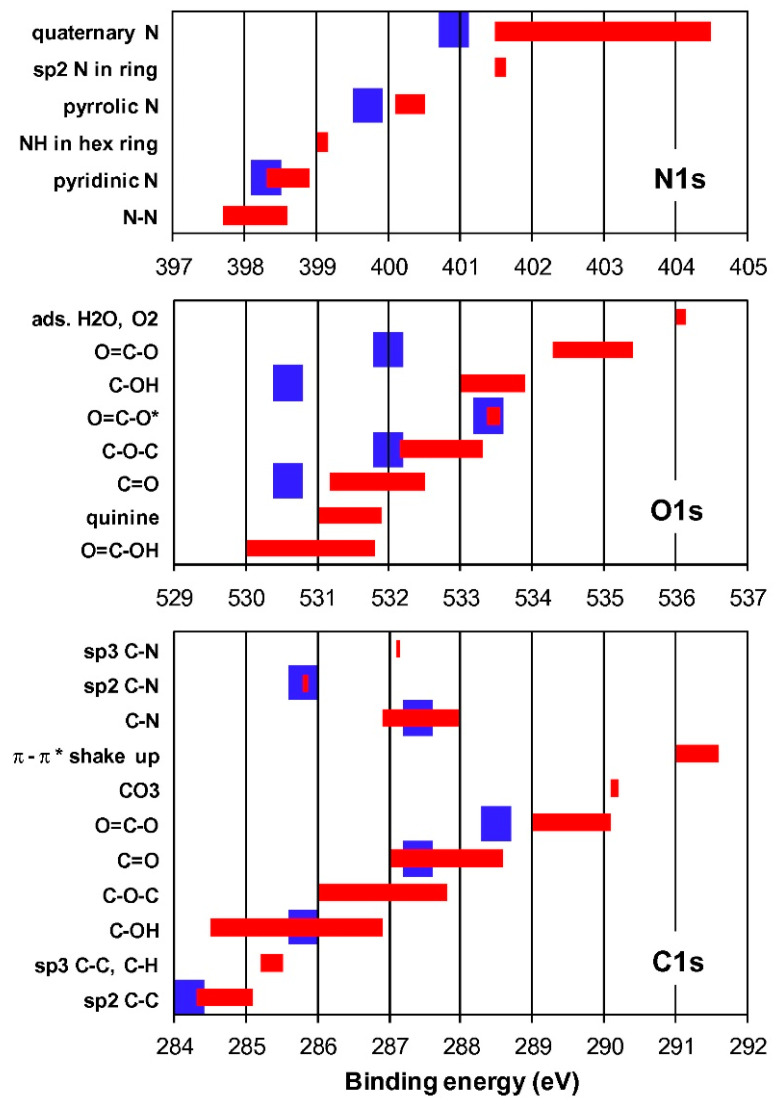
Binding energies of the C1, O1s, and N1s of various carbon nanomaterials as reported by several researchers (red bars) and the ones obtained by Bertoti et al. for multilayered graphene and HOPG treated in nitrogen plasma (blue bars). Bertoti et al. determined peak asymmetry from HOPG reference and strictly limit the peak widths of all subpeaks. Reprinted from Bertoti et al. [7]. Copyright 2020, with permission from Elsevier.

**Figure A13:**
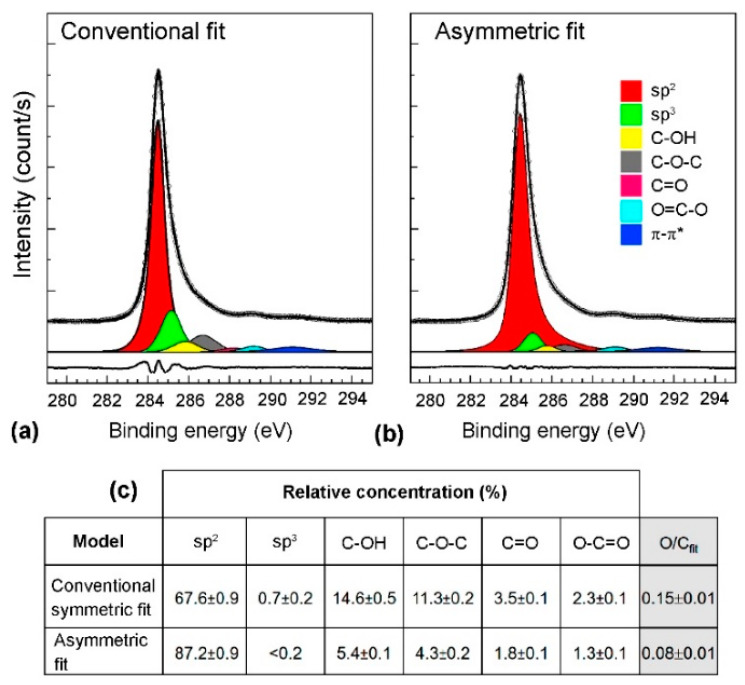
Comparison of the results obtained by the fitting procedure using: (**a**) the conventional symmetric approach and (**b**) another approach by considering sp^2^ asymmetry for graphene sample prepared by CVD. (**c**) When using symmetrical fit as often adopted by researchers, the sp^3^ and oxygen content are overestimated. Reprinted from Kovtun et al. [66]. Copyright 2020, with permission from Elsevier.

**Figure A14:**
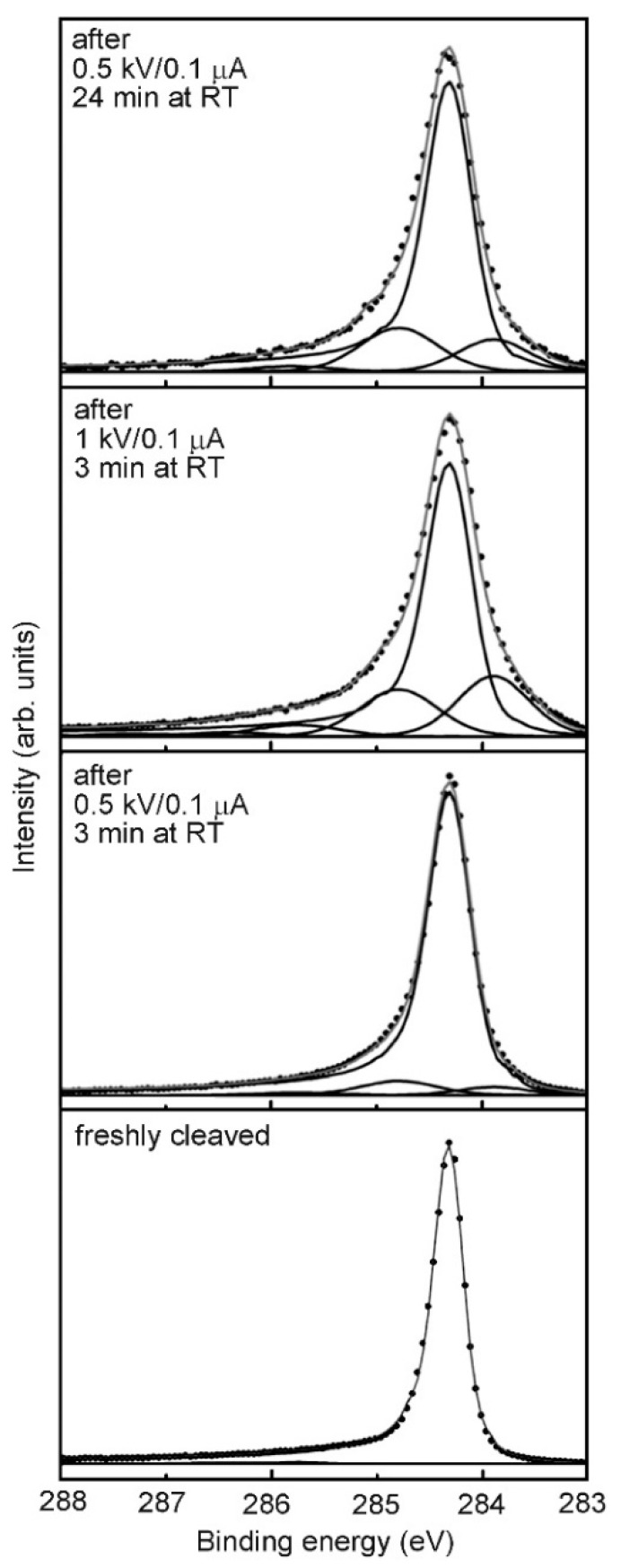
A rise of disordered and defect like carbon peaks in C1s spectra recorded for HOPG exposed to sputtering with the kinetic energy of 150 eV using different accelerating voltage or ion dose (time). Reprinted from Blume et al. [63]. Copyright 2020, with permission from Wiley.

**Figure A15:**
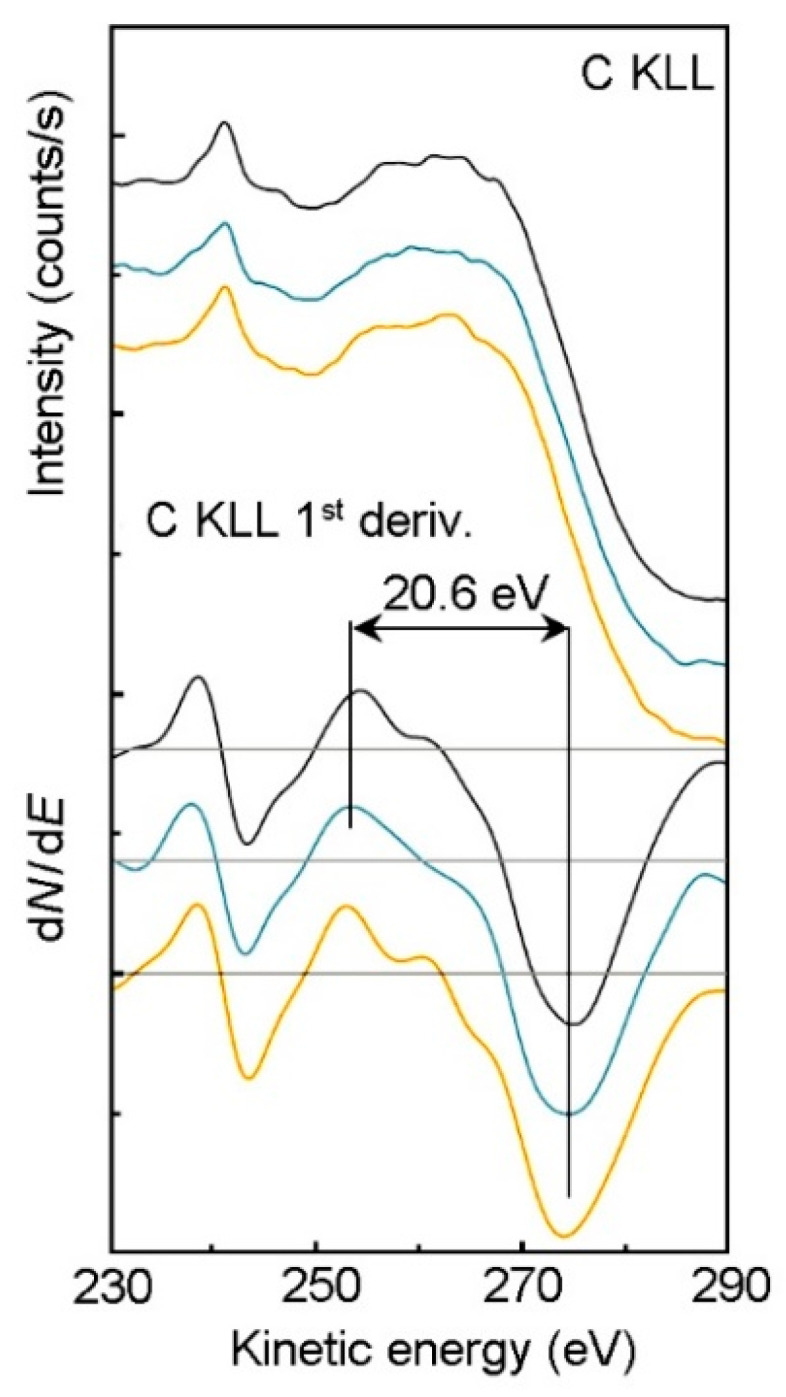
C KLL region in the XPS spectra of N-doped graphene. Reprinted from Bundaleska et al. [14] under the terms of the Creative Commons CC BY license from Springer Nature.

**Figure A16:**
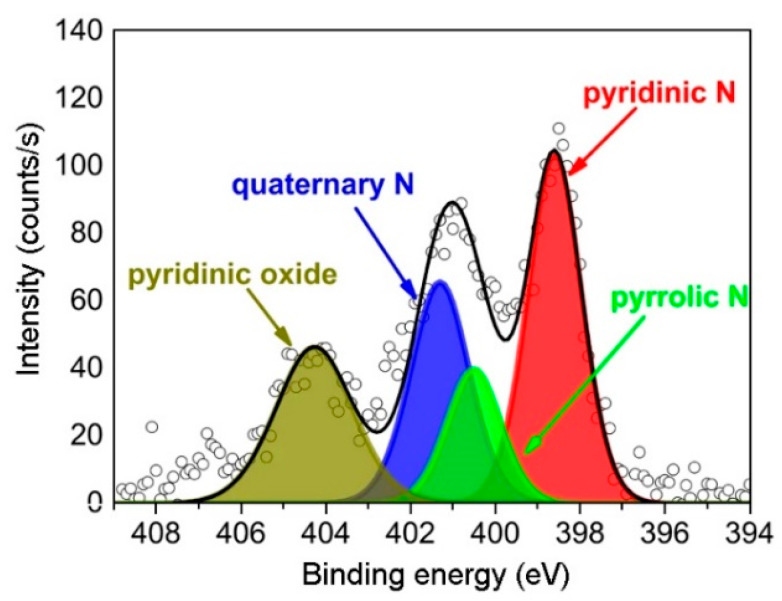
An example of N1s spectra of N-doped graphene. Reprinted from Maddi et al. [71] under the terms of the Creative Commons CC BY license from Springer Nature.

## Abbreviations

CNWs	Carbon nanowalls
ECR	Electron cyclotron resonance
MW	Microwave
RF	Radiofrequency
IC	Inductively coupled
APPJ	Atmospheric-pressure plasma jet
CVD	Chemical vapor deposition
PECVD	Plasma-enhanced chemical vapor deposition
RDE	Rotating disk electrode
CV	Cyclic voltammetry
EIS	Electrochemical impedance spectroscopy
STM	Scanning tunneling microscopy
TEM	Transmission electron spectroscopy
XPS	X-ray photoelectron spectroscopy
AFM	Atomic force microscopy
AAFM	Acoustic atomic force microscopy
ARPES	Angle-resolved photoemission spectroscopy
NEXAFS	Near-edge X-ray absorption fine structure
SAED	Elective area electron diffraction
XRD	X-ray diffraction
SPEM	Scanning photoemission microscopy
UPS	Ultraviolet photoelectron spectroscopy
ARIPES	Angle-resolved inverse photoemission spectroscopy
LEED	Low-energy electron diffraction
WCA	Water contact angle measurements
OES	Optical emission spectroscopy
FTIR	Fourier-transform infrared spectroscopy
ORR	Oxygen reduction reaction

## Appendix A. Raman Spectroscopy of Graphene-Like Materials

Raman spectroscopy is an important technique for surface characterization of carbon-based materials. A typical Raman spectrum of CNWs, graphene and other nanocarbon structures is shown in Figure A1. Raman spectrum of graphene shows two characteristic peaks, i.e., graphitic G band at about ~1580 cm^−1^ and 2D band at about ~2700 cm^−1^. The 2D band is the second-order band (sometimes also marked as G'). The 2D band is always observed for graphene, no matter if a D band is visible or not. The D band (~1350 cm^−1^), which is related to the presence of defects, is usually very weak in graphene samples because of a low number of defects [46].

Raman spectra of various forms of carbon may significantly differ, as shown in Figure A1. The Raman spectra of CNWs are similar to the spectrum of a damaged graphene. Opposite to the spectrum of graphene, they show many second-order modes and disorder-induced features [48]. CNWs and doped graphene with many defects, thus exhibit the significant intensity of D band along with G and 2D bands. With increasing D band intensity, another D' band also appears (~1620 cm^−1^). The D' band is associated with a finite size of graphite crystals and edges of graphene sheets [49]. The D' band is positioned close to the G band and is often observable as a knee on the G band curve because of partial overlapping. Moreover, the position of the G band is shifted upward [50]. An example of this is shown in Figure A2, where a comparison of Raman spectra of graphene, graphene treated in O_2_ plasma, and oxygen-plasma pretreated graphene which was further treated in NH_3_ plasma is shown [51]. The 2D band is much lower in cases of post-treatment in comparison to pristine graphene.

Lucchese et al. [52] investigated the evolution of graphene when exposed to Ar^+^ ion bombardment for various doses from 10^11^ and up to 10^15^ cm^−2^ to induce defects. The energy of ions was 90 eV. While only the G band was observed for the pristine graphene, the D band appeared on irradiated samples, as shown in Figure A3. The intensity of the D band was increasing with increasing ion dose. With increasing D-band intensity, also D’ became apparent, and its intensity was also increasing. For the highest doses above 10^14^ cm^−2^, the intensity of all peaks decreased, and they became wider, indicating amorphization or partial sputtering of graphene despite the rather low kinetic energy of Ar^+^ ions adopted in that study.

The intensity of the D band for CNWs and doped graphene samples with many defects is much higher than the intensity of the G band. Therefore, the intensity ratio of *I*_D_/*I*_G_ is an important parameter [50,53], because the samples containing many defects and nanocrystalline samples will have a high *I*_D_/*I*_G_ ratio. Increasing the *I*_D_/*I*_G_ ratio also causes a broadening of peaks; therefore, FWHM (full width at half maximum) can be correlated with the structural disorder [53]. The *I*_D_/*I*_G_ ratio is inversely proportional to the size of the crystallites *L*_a_, as observed by Tuinstra and Koenig [54]. A large *I*_D_/*I*_G_ ratio is related to smaller crystallites that cause a higher amount of boundary defects. The empirical relation between *L*_a_ and *I*_D_/*I*_G_ was first proposed by Knight and White. The relation was valid only for the laser wavelength of 514.5 nm [55]. This is because the position of D and 2D bands, as well as the ratio *I*_D_/*I*_G_, depends on the excitation laser wavelength [53,56]. Therefore, *I*_D_/*I*_G_ ratio is a function of both the crystallite size *L*_a_ and the laser wavelength:

(A1) $$\frac{I_{D}}{I_{G}}=f\left(L_{a},\lambda \right)$$

An example of the dependence of *I*_D_/*I*_G_ on the crystallite size and wavelength is shown in Figure A4, as reported by Conçado et al. [53]. Conçado et al. [57] improved the Knight-White relation for the determination of *L*_a_ using various excitation laser wavelengths. They proposed the following equation for the crystallite size [57]:

(A2) $$L_{a}\left(\mathrm{nm}\right)=\left(2.4\times 10^{-10}\right)\cdot \lambda^{4}\cdot {\left(\frac{I_{D}}{I_{G}}\right)}^{-1}$$

where *I*_D_/*I*_G_ is the ratio of integrated intensities of D and G bands and *λ* is the excitation laser wavelength (nm). An example of the dependence of *I*_D_/*I*_G_ with *L*_a_ is shown in Figure A5. We can see that Equation (A2) is valid only for *L*_a_ greater than about 4 nm.

As shown by Cançado et al. [53], *I*_D_/*I*_G_ ratio can also be used for the calculation of the density (*N*_D_) of the point defects in graphene with *L_D_* > 10 nm by using the following equation:

(A3) $$N_{D}\left({\mathrm{cm}}^{-2}\right)=\frac{\left(1.8\pm 0.5\right)\times 10^{22}}{\lambda^{4}}{\left(\frac{I_{D}}{I_{G}}\right)}^{}$$

Besides the *I*_D_/*I*_G_ ratio, also *I*_2D_/*I*_G_ and *I*_D_/*I*_D'_ can give additional information. Das et al. [58] have shown that the ratio *I*_2D_/*I*_G_ depended on the doping level. The authors showed clear graphical dependence of the ratio of *I*_2D_/*I*_G_ peak intensities versus the electron concentration. It was also reported that the characteristics of the 2D band depend on the number of graphene layers; however, as reported by Das et al., the *I*_2D_/*I*_G_ should not be used to estimate the number of graphene layers. Nevertheless, in multilayered graphene, changes in width, position, and especially the shape of the peak were observed with the increasing number of layers, as shown in Figure A6 and Figure A7. Also, a change in the ratio of 2D to G is observed (Figure A6). Lee et al. [28] obtained *I*_2D_/*I*_G_ ≈ 2.94 and 0.39 for monolayer and multilayer graphene, respectively. Terasawa et al. have found that *I*_2D_/*I*_G_ was decreasing with increasing nitrogen content, whereas *I*_D_/*I*_G_ was more or less constant [16]. He explained this by the growth of multilayered graphene with smaller grain size. Small *I*_2D_/*I*_G_ ratio and large *I*_D_/*I*_G_ might be, in combination with Equation (A2), associated with a multilayered structure with small grains.

Substantial doping of graphene may induce severe damage of the graphene structure. As graphite is very close in the structure to graphene, we may use a model proposed by Ferrari et al. [50] to investigate modified graphene-like materials. The authors proposed a 3-stage model for the evolution of Raman spectra of graphite during its structural transformation. Stage 1 covers the transformation of graphite to nanocrystalline graphite, stage 2 deals with the transformation of nanocrystalline graphite to low-sp^3^ amorphous carbon, whereas stage 3 deals with the transformation to high-sp^3^ amorphous carbon. The intensity of *I*_D_/*I*_G_ and the G position is changing during this transformation, as illustrated in Figure A8. The correlation observed for stage 1 and 2 is also relevant for CNWs and graphene samples with defects. We can see that in stage 1, both the position of G and *I*_D_/*I*_G_ are increasing, whereas, in stage 2, the opposite is observed, i.e., they both decrease.

Raman spectra of nanocrystalline graphene samples with a low contribution of some sp^3^ carbon will thus show a shift of G band back to lower values, a decrease of the ratio *I*_D_/*I*_G_, and a loss of the second-order features [53]. Further investigation of Raman characteristics of the transition between stages 1 to 2 was performed by Eckmann et al. [60]. They investigated the intensity of D, G, D' and 2D bands as well as the *I*_D_/*I*_D'_ ratio of oxidized graphene versus plasma treatment time and observed the transition from stage 1 to stage 2 (Figure A9). In stage 1, there was no difference when plotting the variation of the peak intensity or integrated peak areas, because the trend was the same. However, in stage 2, different behavior of the maximum intensity or integrated area with treatment time was observed because of compensation of intensity and increment of FWHM. This indicates that in stage 1, it is not so important if the ratio is calculated from maximum intensities or integrated areas. However, in stage 2, peak areas are more representative. The *I*_D_/*I*_G_ ratio in stages 1 or 2 may be the same, but not FWHM. Therefore, FWHM, which is much larger in stage 2, can give additional information on the stage [53]. According to Figure A9 we can also observe significant trends of various bands in stage 1 or 2, which are summarized in Table A1. Eckmann also found that in stage 1, D and D' are proportional, and the proportionality factor depends on the type of defects in the sample [60]. For sp^3^ defects *I*_D_/*I*_D'_ ≈ 13, for vacancy-like defects *I*_D_/*I*_D'_ ≈ 7, and for boundary defects *I*_D_/*I*_D'_ ≈ 3.5.

**Table A1:** Behavior of Raman D, D', 2D, and G bands in stages 1 and 2, according to Eckmann [60].

	D Band	D’ Band	2D Band	G Band
Stage 1	increase	increase	decrease	~constant
Stage 2	decrease	increase	sharp decrease	intensity decrease, area increase

All the above-cited observations are summarized in Figure A10. According to Ferrari [50], the transition between stages 1 and 2 for graphite is observed at *I*_D_/*I*_G_ ≈ 2; however, Eckmann et al. [60] observed this transition at *I*_D_/*I*_G_ ≈ 4 for oxidized graphene obtained by plasma treatment at several exposure times.

## Appendix B. X-ray Photoelectron Spectroscopy of Graphene-Like Materials

XPS is another technique that can give additional information regarding the quality of carbon nanostructures and the presence of heteroatoms as well as the type of functional groups that were formed during the modification of graphene samples. Carbon C1s peak of a pure graphite-like sample with a high sp^2^ content, such as graphene, will have a strong asymmetric peak, described by its asymmetry factor α. Furthermore, several π-type shake-up satellite features are present and positioned several eV at higher binding energies from the main C1s peak (Figure A11). Satellite features for graphite were in detail described by Leiro et al. [61]. Also, the presence of any functional group will influence the shape of the C1s peak.

The main C1s peak of graphite-like materials is positioned at approximately ~284.4 eV [62]. The materials with high sp^3^ content will have a more symmetric peak, which is also slightly shifted to higher energy. Modified (doped) graphene samples will thus have a contribution of both asymmetric sp^2^ component (graphitic carbon) and symmetric sp^3^ component (defects) as well as the presence of additional symmetric peaks because of the covalently linked functional groups. This combination of asymmetric and symmetric components complicates the fitting of the C1s spectra, which is not straightforward. Therefore, the determination of sp^2^/sp^3^ contributions as regularly performed in published literature is often questionable. Furthermore, the spectrum C1s belonging to the sp^2^ component should be fitted with an asymmetric peak, whereas other subcomponents with symmetric peaks. However, the asymmetry is often neglected in the scientific literature. The asymmetry of the sp^2^ component shape can be determined on a reference sample. As a reference, often HOPG (highly-oriented-pyrolytic graphite) is used; however, we should be aware that the asymmetry of the sample with defects may change [63].

As already mentioned, many authors often simplify the fitting procedure by using only symmetric peaks, often taking into account also different peak widths. Furthermore, satellite features on the high-binding energy side of the spectrum several eV from the main C1s peak are sometimes not considered. It has been shown that the spectra of satellite features of graphite, diamond, and amorphous carbon differ in the shape and position [64] and even for the same material such as HOPG, depending on the sample preparation [63]. By neglecting the peak asymmetry and broad satellite features, the authors often fit a high-binding energy side of the spectrum with carbon-oxygen groups, whose presence is therefore questionable and the number of these groups and their concentration is overestimated. Furthermore, as shown by Bertoti et al. [7], the complexity caused by the peak asymmetry and its neglecting in the fitting procedure, the presence of various oxygen and nitrogen bonds, interaction among these moieties, etc. leads to significant scattering in the binding energy values of assigned peaks as shown in Figure A12.

A good example of the overestimation of the sp^3^ and oxygen content because of using conventional symmetric functions was given by Kovtun et al. In Figure A13 is shown a comparison of the fitting of the C1s peak of graphene using a conventional symmetric and asymmetric approach. We can see that the sp^3^ content, as well as oxygen content, were significantly overestimated on account of lower sp^2^ content if the symmetric fitting was used.

Susi et al. [62] presented a review of reported binding energies of the main C1s peak, their FWHM (full-width at half maximum), and the asymmetry index for various graphitic carbon nanomaterials. They also reported that a shift of the C 1s peak position to higher binding energies was observed upon nitrogen doping. The C1s shift was in the range between 0.1 to 0.4 eV. They also pointed out the problems of the broad range of the binding energies that were reported for various N1s configurations.

Blume et al. [63] presented an overview of problems associated with XPS analysis of carbon samples and how to identify and distinguish carbon species from their oxygen functionality. The authors showed how C1s spectra of HOPG, mono- and few-layer graphene, graphene flakes, graphene oxide, and single- and multi-wall carbon nanotubes differ in the binding energy, width, and shape of the peak, although the samples are “similar”. Many factors can have an impact on the line shape of the peak and its position, like surface contamination, the presence of disorder carbon, layer-to-layer interactions, and substrate interactions, topographic effects, etc. Fujimoto et al. [65] reported that also charging may significantly influence the assignment of the sp^3^ peaks because there are some reports where sp^3^ was found at lower binding energy than sp^2^. However, as shown by Blume et al. [63], sputtering of HOPG may result in broadening of the C1s on the high- as well as at low-binding energy side pointing out the different nature of these defects. These two different contributions can be assigned to disordered carbon and defect-like carbon. An example of the influence of disordered carbon and defects on the shape of C1s spectra is shown in Figure A14. This figure shows oxygen-free HOPG after sputtering using different sputter parameters to induce defects. With increasing accelerating voltage or ion dose, a broadening appears on the high binding-energy side (disordered C) as well as a shoulder at the low binding-energy side (defects). A similar observation was reported by other authors as well [14].

Because of the uncertainties in the determination of the sp^2^ and sp^3^ content in the C1s spectra, some authors tried to use the so-called D-parameter, which is defined as the energy distance between the minimum and maximum of the first derivatives of Auger C KLL spectra (1190–1250 eV) [65,67,68]. The higher D-parameters are related to a higher amount of sp^2^. The D-parameter for graphite is thus larger than for diamond, i.e., D ≈ 21 and D ≈ 14 for graphite and diamond, respectively [67,69]. The major problem is associated with defining the right maximum/minimum of the smoothed spectra. Bundaleska et al. [14] measured the C KLL spectra for N-doped graphene (Figure A15) after applying smoothing with 11 successive moving averages and obtained the D-value of 20.6 eV, which is close to the value as reported by Kaciulis and Mezzi for graphite [67,69].

In the case of doping of graphitic samples with nitrogen, N1s is the most important XPS peak of investigation. As already mentioned in the introduction and shown in Figure 1, nitrogen in N-doped graphene samples can be found in various configurations like pyridinic, pyrrolic, graphitic nitrogen, and oxidized nitrogen groups. An example of the nitrogen N1s spectrum with subcomponents corresponding to different nitrogen configurations in N-doped graphene is shown in Figure A16. The reported range of the binding-energy (BE) values for various nitrogen configurations are shown in Table A2. Yamada et al. [70] reported calculated values for the peak shifts and their FWHMs. They found that the average shift of the BE values of N1s reported by other authors is quite well correlated with their calculations for some functional groups, including pyridinic, pyrrolic, and quaternary nitrogen. They also reported that functional groups originating from edges have lower FWHM than those originating from the basal planes. Zhang et al. [13] noticed a shift of the N1s peak position with the growth temperature of N-doped graphene synthesized at, what was explained by different local environment of N atoms because of formation of defects.

**Table A2:** Reported values of binding energies of various nitrogen configurations.

Pyridinic (N1)	Pyrrolic (N2)	Graphitic/Quaternary (N3)	Amine	Oxidized Forms	Ref.
398.3	399.7	400.9			[7]
398.7	400.2	402.3			[14]
398.6	400.1	401.1			[33]
398.9	400.2	401.7			[12]
398.7	400.1	401.8			[31]
398.5–398.6	399.6–399.8	401.1			[18]
398.4	399.9	401.2			[72]
398.5	400.1	401.5			[23]
398.2	400.1	401.7	399.0		[28]
~398	~400	~401			[32]
398.7	400.3	401.4	~400.3	402–405	[19]
398.9	400.1	401.1		402.6	[11]
398.7	400.3	401.2		402.8	[43]
398.5	399.9	401			[41]
399.4	/	401.2			[15]
398.9	400.1	401.5			[42]
398.3	400.5	/			[4]
398.9	399.6	401.2			[40]
398.4	/	/			[20]
/	/	/	399.6		[21]
398.2	400.3	401.5			[44]
/	399.3	/			[34]
398.3–398.4	399.6–399.9	400.9–402.4		405.6–406.1	[13]

For graphene samples treated in ammonia plasma, sometimes also amino groups were reported (Table A2); however, because of its overlapping pyrrolic group, its presence cannot be unambiguously confirmed. An exception is a publication by Baraket et al. [27], who proved their presence by chemical derivatization; however, no details were given.

Despite all the problems regarding XPS characterization, as stated above, XPS is still an indispensable method for the characterization of graphene-like materials; however, it is sometimes difficult to compare quantitative results from the literature because the authors use a different way of presenting their results. This is evident from the review of the N-doped graphene synthesis procedures summarized in Table 1 and Table 2, and particularly in Table 3. We also noticed that nitrogen concentration is often given in “percentage”; however, sometimes it is not clear if the reported value is N/C ratio or N concentration in atomic percent. Furthermore, the concentration of other elements is often not stated, neither details about fitting procedure described.

## References

[1] Evlashin S.A., Fedorov F.S., Dyakonov P.V., Maksimov Y.M., Pilevsky A.A., Maslakov K.I., Kuzminova Y.O., Mankelevich Y.A., Voronina E.N., Dagesyan S.A. (2020). Role of nitrogen and oxygen in capacitance formation of carbon nanowalls. J. Phys. Chem. Lett..

[2] Xu H., Ma L., Jin Z. (2018). Nitrogen-doped graphene: Synthesis, characterizations and energy applications. J. Energy Chem..

[3] Mahmood N., Zhang C.Z., Yin H., Hou Y.L. (2014). Graphene-based nanocomposites for energy storage and conversion in lithium batteries, supercapacitors and fuel cells. J. Mater. Chem. A.

[4] Qu L., Liu Y., Baek J.-B., Dai L. (2010). Nitrogen-doped graphene as efficient metal-free electrocatalyst for oxygen reduction in fuel cells. ACS Nano.

[5] Vesel A., Zaplotnik R., Primc G., Mozetič M. (2019). Synthesis of vertically oriented graphene sheets or carbon nanowalls—Review and challenges. Materials.

[6] Schiros T., Nordlund D., Pálová L., Prezzi D., Zhao L., Kim K.S., Wurstbauer U., Gutiérrez C., Delongchamp D., Jaye C. (2012). Connecting dopant bond type with electronic structure in N-doped graphene. Nano Lett..

[7] Bertóti I., Mohai M., László K. (2015). Surface modification of graphene and graphite by nitrogen plasma: Determination of chemical state alterations and assignments by quantitative X-ray photoelectron spectroscopy. Carbon.

[8] Boas C.R.S.V., Focassio B., Marinho E., Larrude D.G., Salvadori M.C., Leão C.R., dos Santos D.J. (2019). Characterization of nitrogen doped graphene bilayers synthesized by fast, low temperature microwave plasma-enhanced chemical vapour deposition. Sci. Rep..

[9] Usachov D., Vilkov O., Grüneis A., Haberer D., Fedorov A., Adamchuk V.K., Preobrajenski A.B., Dudin P., Barinov A., Oehzelt M. (2011). Nitrogen-doped graphene: Efficient growth, structure, and electronic properties. Nano Lett..

[10] Zhao L., He R., Rim K.T., Schiros T., Kim K.S., Zhou H., Gutiérrez C., Chockalingam S.P., Arguello C.J., Pálová L. (2011). Visualizing individual nitrogen dopants in monolayer graphene. Science.

[11] Singh G., Sutar D.S., Botcha V.D., Narayanam P.K., Talwar S.S., Srinivasa R.S., Major S.S. (2013). Study of simultaneous reduction and nitrogen doping of graphene oxide Langmuir-Blodgett monolayer sheets by ammonia plasma treatment. Nanotechnol..

[12] Cho H.J., Kondo H., Ishikawa K., Sekine M., Hiramatsu M., Hori M. (2014). Effects of nitrogen plasma post-treatment on electrical conduction of carbon nanowalls. Jpn. J. Appl. Phys..

[13] Zhang J., Zhao C., Liu N., Zhang H., Liu J., Fu Y.Q., Guo B., Wang Z., Lei S., Hu P. (2016). Tunable electronic properties of graphene through controlling bonding configurations of doped nitrogen atoms. Sci. Rep..

[14] Bundaleska N., Henriques J., Abrashev M., Botelho do Rego A.M., Ferraria A.M., Almeida A., Dias F.M., Valcheva E., Arnaudov B., Upadhyay K.K. (2018). Large-scale synthesis of free-standing N-doped graphene using microwave plasma. Sci. Rep..

[15] Tatarova E., Dias A., Henriques J., Abrashev M., Bundaleska N., Kovacevic E., Bundaleski N., Cvelbar U., Valcheva E., Arnaudov B. (2017). Towards large-scale in free-standing graphene and N-graphene sheets. Sci. Rep..

[16] Terasawa T.-O., Saiki K. (2012). Synthesis of nitrogen-doped graphene by plasma-enhanced chemical vapor deposition. Jpn. J. Appl. Phys..

[17] Kumar A., Voevodin A.A., Paul R., Altfeder I., Zemlyanov D., Zakharov D.N., Fisher T.S. (2013). Nitrogen-doped graphene by microwave plasma chemical vapor deposition. Thin Solid Films.

[18] Evlashin S.A., Maksimov Y.M., Dyakonov P.V., Pilevsky A.A., Maslakov K.I., Mankelevich Y.A., Voronina E.N., Vavilov S.V., Pavlov A.A., Zenova E.V. (2019). N-doped carbon nanowalls for power sources. Sci. Rep..

[19] McClure J.P., Thornton J.D., Jiang R.Z., Chu D., Cuomo J.J., Fedkiw P.S. (2012). Oxygen reduction on metal-free nitrogen-doped carbon nanowall electrodes. J. Electrochem. Soc..

[20] Yen H.F., Horng Y.Y., Hu M.S., Yang W.H., Wen J.R., Ganguly A., Tai Y., Chen K.H., Chen L.C. (2015). Vertically aligned epitaxial graphene nanowalls with dominated nitrogen doping for superior supercapacitors. Carbon.

[21] Zhao C.X., Zhang Y., Deng S.Z., Xu N.S., Chen J. (2016). Surface nitrogen functionality for the enhanced field emission of free-standing few-layer graphene nanowalls. J. Alloys Compd..

[22] Achour A., Solaymani S., Vizireanu S., Baraket A., Vesel A., Zine N., Errachid A., Dinescu G., Pireaux J.J. (2019). Effect of nitrogen configuration on carbon nanowall surface: Towards the improvement of electrochemical transduction properties and the stabilization of gold nanoparticles. Mater. Chem. Phys..

[23] Jeong H.M., Lee J.W., Shin W.H., Choi Y.J., Shin H.J., Kang J.K., Choi J.W. (2011). Nitrogen-doped graphene for high-performance ultracapacitors and the importance of nitrogen-doped sites at basal planes. Nano Lett..

[24] McManus J.B., Hennessy A., Cullen C.P., Hallam T., McEvoy N., Duesberg G.S. (2017). Controlling defect and dopant concentrations in graphene by remote plasma treatments. Phys. Status Solidi B-Basic Solid State Phys..

[25] Lin Y.-P., Ksari Y., Prakash J., Giovanelli L., Valmalette J.-C., Themlin J.-M. (2014). Nitrogen-doping processes of graphene by a versatile plasma-based method. Carbon.

[26] Zeng J.J., Lin Y.J. (2014). Tuning the work function of graphene by nitrogen plasma treatment with different radio-frequency powers. Appl. Phys. Lett..

[27] Baraket M., Stine R., Lee W.K., Robinson J.T., Tamanaha C.R., Sheehan P.E., Walton S.G. (2012). Aminated graphene for DNA attachment produced via plasma functionalization. Appl. Phys. Lett..

[28] Lee B.-J., Cho S.-C., Jeong G.-H. (2015). Atmospheric pressure plasma treatment on graphene grown by chemical vapor deposition. Curr. Appl. Phys..

[29] Santhosh N.M., Filipic G., Kovacevic E., Jagodar A., Berndt J., Strunskus T., Kondos H., Hori M., Tatarova E., Cvelbar U. (2020). N-graphene nanowalls via plasma nitrogen incorporation and substitution: The experimental evidence. Nanomicro Lett..

[30] Manojkumar P.A., Krishna N.G., Mangamma G., Albert S.K. (2019). Understanding the structural and chemical changes in vertical graphene nanowalls upon plasma nitrogen ion implantation. Phys. Chem. Chem. Phys..

[31] Deng D., Pan X., Yu L., Cui Y., Jiang Y., Qi J., Li W.-X., Fu Q., Ma X., Xue Q. (2011). Toward N-doped graphene via solvothermal synthesis. Chem. Mater..

[32] Lu Y.-F., Lo S.-T., Lin J.-C., Zhang W., Lu J.-Y., Liu F.-H., Tseng C.-M., Lee Y.-H., Liang C.-T., Li L.-J. (2013). Nitrogen-doped graphene sheets grown by chemical vapor deposition: Synthesis and influence of nitrogen impurities on carrier transport. ACS Nano.

[33] Amano T., Kondo H., Takeda K., Ishikawa K., Hiramatsu M., Sekine M., Hori M. (2018). Oxygen reduction reaction properties of nitrogen-incorporated nanographenes synthesized using in-liquid plasma from mixture of ethanol and iron phthalocyanine. Jpn. J. Appl. Phys..

[34] Luo Z., Lim S., Tian Z., Shang J., Lai L., MacDonald B., Fu C., Shen Z., Yu T., Lin J. (2011). Pyridinic N doped graphene: Synthesis, electronic structure, and electrocatalytic property. J. Mater. Chem..

[35] Sui Y., Zhu B., Zhang H., Shu H., Chen Z., Zhang Y., Zhang Y., Wang B., Tang C., Xie X. (2015). Temperature-dependent nitrogen configuration of N-doped graphene by chemical vapor deposition. Carbon.

[36] Wei D., Liu Y., Wang Y., Zhang H., Huang L., Yu G. (2009). Synthesis of N-doped graphene by chemical vapor deposition and its electrical properties. Nano Lett..

[37] Dias A., Bundaleski N., Tatarova E., Dias F.M., Abrashev M., Cvelbar U., Teodoro O.M.N.D., Henriques J. (2016). Production of N-graphene by microwave N_2_-Ar plasma. J. Phys. D Appl. Phys..

[38] Lin Y.C., Lin C.Y., Chiu P.W. (2010). Controllable graphene N-doping with ammonia plasma. Appl. Phys. Lett..

[39] Kato T., Jiao L., Wang X., Wang H., Li X., Zhang L., Hatakeyama R., Dai H. (2011). Room-temperature edge functionalization and doping of graphene by mild plasma. Small.

[40] Yang W., Xu X., Tu Z., Li Z., You B., Li Y., Raj S.I., Yang F., Zhang L., Chen S. (2015). Nitrogen plasma modified CVD grown graphene as counter electrodes for bifacial dye-sensitized solar cells. Electrochim. Acta.

[41] Sim U., Yang T.-Y., Moon J., An J., Hwang J., Seo J.-H., Lee J., Kim K.Y., Lee J., Han S. (2013). N-doped monolayer graphene catalyst on silicon photocathode for hydrogen production. Energy Environ. Sci..

[42] Wang Y., Shao Y., Matson D.W., Li J., Lin Y. (2010). Nitrogen-doped graphene and its application in electrochemical biosensing. ACS Nano.

[43] Shao Y., Zhang S., Engelhard M.H., Li G., Shao G., Wang Y., Liu J., Aksay I.A., Lin Y. (2010). Nitrogen-doped graphene and its electrochemical applications. J. Mater. Chem..

[44] Zhang C., Fu L., Liu N., Liu M., Wang Y., Liu Z. (2011). Synthesis of nitrogen-doped graphene using embedded carbon and nitrogen sources. Adv. Mater..

[45] Mozetic M., Vesel A., Stoica S.D., Vizireanu S., Dinescu G., Zaplotnik R. (2015). Oxygen atom loss coefficient of carbon nanowalls. Appl. Surf. Sci..

[46] Ferrari A.C., Meyer J.C., Scardaci V., Casiraghi C., Lazzeri M., Mauri F., Piscanec S., Jiang D., Novoselov K.S., Roth S. (2006). Raman spectrum of graphene and graphene layers. Phys. Rev. Lett..

[47] Hiramatsu M., Kondo H., Hori M., Ebrahimi F. (2015). Nanoplatform Based on Vertical Nanographene. Graphene-New Trends and Developments.

[48] Dresselhaus M.S., Jorio A., Hofmann M., Dresselhaus G., Saito R. (2010). Perspectives on carbon nanotubes and graphene Raman spectroscopy. Nano Lett..

[49] Hiramatsu M., Hori M. (2010). Carbon nanowalls: Synthesis and Emerging Applications.

[50] Ferrari A.C., Robertson J. (2000). Interpretation of Raman spectra of disordered and amorphous carbon. Phys. Rev. B.

[51] McEvoy N., Nolan H., Kumar N.A., Hallam T., Duesberg G.S. (2013). Functionalisation of graphene surfaces with downstream plasma treatments. Carbon.

[52] Lucchese M.M., Stavale F., Ferreira E.H.M., Vilani C., Moutinho M.V.O., Capaz R.B., Achete C.A., Jorio A. (2010). Quantifying ion-induced defects and Raman relaxation length in graphene. Carbon.

[53] Cançado L.G., Jorio A., Ferreira E.H.M., Stavale F., Achete C.A., Capaz R.B., Moutinho M.V.O., Lombardo A., Kulmala T.S., Ferrari A.C. (2011). Quantifying defects in graphene via Raman spectroscopy at different excitation energies. Nano Lett..

[54] Tuinstra F., Koenig J.L. (1970). Raman spectrum of graphite. J. Chem. Phys..

[55] Knight D.S., White W.B. (2011). Characterization of diamond films by Raman spectroscopy. J. Mater. Res..

[56] Ferrari A.C. (2007). Raman spectroscopy of graphene and graphite: Disorder, electron–phonon coupling, doping and nonadiabatic effects. Solid State Commun..

[57] Cançado L.G., Takai K., Enoki T., Endo M., Kim Y.A., Mizusaki H., Jorio A., Coelho L.N., Magalhães-Paniago R., Pimenta M.A. (2006). General equation for the determination of the crystallite size La of nanographite by Raman spectroscopy. Appl. Phys. Lett..

[58] Das A., Pisana S., Chakraborty B., Piscanec S., Saha S.K., Waghmare U.V., Novoselov K.S., Krishnamurthy H.R., Geim A.K., Ferrari A.C. (2008). Monitoring dopants by Raman scattering in an electrochemically top-gated graphene transistor. Nature Nanotechnol..

[59] Malard L.M., Pimenta M.A., Dresselhaus G., Dresselhaus M.S. (2009). Raman spectroscopy in graphene. Phys. Rep..

[60] Eckmann A., Felten A., Mishchenko A., Britnell L., Krupke R., Novoselov K.S., Casiraghi C. (2012). Probing the nature of defects in graphene by Raman spectroscopy. Nano Lett..

[61] Leiro J.A., Heinonen M.H., Laiho T., Batirev I.G. (2003). Core-level XPS spectra of fullerene, highly oriented pyrolitic graphite, and glassy carbon. J. Electron Spectros. Relat. Phenom..

[62] Susi T., Pichler T., Ayala P. (2015). X-ray photoelectron spectroscopy of graphitic carbon nanomaterials doped with heteroatoms. Beilstein J. Nanotechnol..

[63] Blume R., Rosenthal D., Tessonnier J.-P., Li H., Knop-Gericke A., Schlögl R. (2015). Characterizing graphitic carbon with x-ray photoelectron spectroscopy: A step-by-step approach. ChemCatChem.

[64] Haq S., Tunnicliffe D.L., Sails S., Savage J.A. (1996). Assessment of nondiamond carbon levels present in chemical vapor deposited diamond by analysis of the plasmon loss features of x-ray photoelectron spectra. Appl. Phys. Lett..

[65] Fujimoto A., Yamada Y., Koinuma M., Sato S. (2016). Origins of sp^3^C peaks in C1s X-ray photoelectron spectra of carbon materials. Anal. Chem..

[66] Kovtun A., Jones D., Dell’Elce S., Treossi E., Liscio A., Palermo V. (2019). Accurate chemical analysis of oxygenated graphene-based materials using X-ray photoelectron spectroscopy. Carbon.

[67] Mezzi A., Kaciulis S. (2010). Surface investigation of carbon films: From diamond to graphite. Surf. Interface Anal..

[68] Theodosiou A., Spencer B.F., Counsell J., Jones A.N. (2020). An XPS/UPS study of the surface/near-surface bonding in nuclear grade graphites: A comparison of monatomic and cluster depth-profiling techniques. Appl. Surf. Sci..

[69] Kaciulis S., Mezzi A., Calvani P., Trucchi D.M. (2014). Electron spectroscopy of the main allotropes of carbon. Surf. Interface Anal..

[70] Yamada Y., Kim J., Matsuo S., Sato S. (2014). Nitrogen-containing graphene analyzed by X-ray photoelectron spectroscopy. Carbon.

[71] Maddi C., Bourquard F., Barnier V., Avila J., Asensio M.-C., Tite T., Donnet C., Garrelie F. (2018). Nano-architecture of nitrogen-doped graphene films synthesized from a solid CN source. Sci. Rep..

[72] Huang L., Cao Y., Diao D. (2019). N-doped graphene sheets induced high electrochemical activity in carbon film. Appl. Surf. Sci..

